# Gene Editing by Co-Transformation of TALEN and Chimeric RNA/DNA Oligonucleotides on the Rice *OsEPSPS* Gene and the Inheritance of Mutations

**DOI:** 10.1371/journal.pone.0122755

**Published:** 2015-04-09

**Authors:** Mugui Wang, Yujun Liu, Cuicui Zhang, Jianping Liu, Xin Liu, Liangchao Wang, Wenyi Wang, Hao Chen, Chuchu Wei, Xiufen Ye, Xinyuan Li, Jumin Tu

**Affiliations:** Institute of Crop Sciences, College of Agriculture and Biotechnology, Zhejiang University, Hangzhou, Zhejiang Province, China; Institute of Crop Sciences, CHINA

## Abstract

Although several site-speciﬁc nucleases (SSNs), such as zinc-ﬁnger nucleases (ZFNs), transcription activator-like effector nucleases (TALENs), and the clustered regularly interspaced short palindromic repeat (CRISPR)/Cas, have emerged as powerful tools for targeted gene editing in many organisms, to date, gene targeting (GT) in plants remains a formidable challenge. In the present study, we attempted to substitute a single base *in situ *on the rice *OsEPSPS *gene by co-transformation of TALEN with chimeric RNA/DNA oligonucleotides (COs), including different strand composition such as RNA/DNA (C1) or DNA/RNA (C2) but contained the same target base to be substituted. In contrast to zero GT event obtained by the co-transformation of TALEN with homologous recombination plasmid (HRP), we obtained one mutant showing target base substitution although accompanied by undesired deletion of 12 bases downstream the target site from the co-transformation of TALEN and C1. In addition to this typical event, we also obtained 16 mutants with different length of base deletions around the target site among 105 calli lines derived from transformation of TALEN alone (4/19) as well as co-transformation of TELAN with either HRP (5/30) or C1 (2/25) or C2 (5/31). Further analysis demonstrated that the homozygous gene-edited mutants without foreign gene insertion could be obtained in one generation. The induced mutations in transgenic generation were also capable to pass to the next generation stably. However, the genotypes of mutants did not segregate normally in T1 population, probably due to lethal mutations. Phenotypic assessments in T1 generation showed that the heterozygous plants with either one or three bases deletion on target sequence, called d_1_ and d_3_, were more sensitive to glyphosate and the heterozygous d1 plants had significantly lower seed-setting rate than wild-type.

## Introduction

In the past few years, the development of site-specific nucleases (SSNs), such as zinc-finger nucleases (ZFNs), transcription activator-like effector nucleases (TALENs), and the clustered regularly interspaced short palindromic repeat (CRISPR)/Cas, have applied successfully in genome modifications in different species [1–8]. However, in higher plants, most of the above applications resulted in gene knockout. Target gene insertion or replacement (both termed gene targeting here, GT) is still far from common practice. So far, a few successful GT events using TALEN or CRISPR/Cas in plants such as tobacco [3,7] and rice [6] have been reported, but the explants used in all of these studies were protoplasts. Compared to mammalian and human cells, one challenge for GT in plants is the efficient delivery of SSNs-containing vectors and donor DNA molecules into the cells [3]. For many plants, the efficiency of transformation of these cells or tissues by *Agrobacterium tumefaciens* or physical means such as biolistic bombardment is low due to existence of cell wall barrier [9]. To overcome this difficulty, an in planta GT system [10,11] and a geminivirus-based strategy [12,13] were developed. GT events can occur in the whole plant during its lifecycle in the former system, while in the latter one the GT events can be also enhanced significantly by large replication of the donor templates via geminivirus. Recently, a co-transformation strategy was also successfully applied in target gene knock-in in immature embryos of bread wheat with TALEN and short single stranded DNAs (ssDNAs) [14]. However, the efficiency was sill low, with one GT event obtained in 69 regenerated transgenic plants for one target site and one in 39 for another target locus. As an alternative, we proposed that using chimeric RNA/DNA oligonucleotides (COs) in the co-transformation strategy might has more advantages than ssDNAs in the case of modifications for one to several bases.

COs is a kind of short self-complementary chimeras consisting of RNA and DNA residues, capped at both ends by sequences which fold into a hairpin [15–17]. Such a structure confers COs capably avoiding destabilization or destruction by cellular helicases or exonucleases. An additional feature in the design of COs is modification of the RNA residues by 2'-O-methylation to render the oligonucleotides resistant to the RNase H activity present in mammalian cells [15–17]. COs contains a DNA ‘mutator’ region of five nucleotides complementary to the target site, which includes one or more bases that do not pair with the target sequence, and can be introduced into the target via the host cell’s mismatch repair function [16,17]. Due to the high specificity and as “clean” donors that can not insert into the host genome, COs have been successfully used for conducting site-specific base changes in both episomal and chromosomal target genes in human and mammalian cells [16–19]. COs also benefit for direct gene modification in plants. Compared to conventional plasmid donors, the low molecular weight of COs (generally 68 nucleotides) make it possible to be introduced into partial cells with a large number of molecules by biolistic bombardment. To date, successful gene modifications with COs independently have been reported in maize [20,21], tobacco [22], rice [23] and wheat [24].

However, because of the inactivity of COs, gene modification mediated by this molecule is only obtained by the random encounter between COs and the target sequence. The low efficiency (approximately 1×10^–4^) of this strategy limits its applicability in plants. Since the double strands breaks (DSBs) introduced on target loci could strongly increase the frequency of homologous recombination (HR) [25], we proposed that DSB might also promote the efficiency of GT with COs. The high activity of DSB-inducing nucleases might partially complement the “inactivity” of the COs. Previous study showed that a single base transition of C^317^-T within the *OsEPSPS*, causing a change of proline-106 to leucine (P106L) in the protein sequence, exhibited glyphosate resistance in EPSPS-deficient *Escherichia coli* (*E*. *coli*) strain, AB2829 [26]. In the present study, we attempted to achieve the similar glyphosate resistance in rice by direct substitution of the target base C^317^ to T in the coding sequence of *OsEPSPS* via TALEN assisted with COs molecule. Therefore, a TALEN construct was designed to induce a DSB near the C^317^, and three COs molecules, including one with typical length of 68 nt, called C0 and two structurally improved ones with longer length of 88 nt, called C1 and C2, respectively, were designed to *in situ* substitute C^317^ to T. In addition to separate transformation of TALEN or COs, the two structurally improved C1 and C2 were also co-transformed into the rice with TALEN, respectively. As an alternative, a homologous recombination plasmid (HRP) that contains a modified 898 bp *OsEPSPS* homologous fragment was also co-transformed with TALEN as a GT donor since the homologous fragments were usually used as template for repairing the DSB in GT process [3,4,27].

We reported here the results obtained via above experimental designing and procedures. We also discussed the possibility of GT with COs as donor in the co-transformation strategy and summarized the possible variations in the mutation flow.

## Materials and Methods

### Construction of COs, TALEN, and HRP

**Fig 1:**
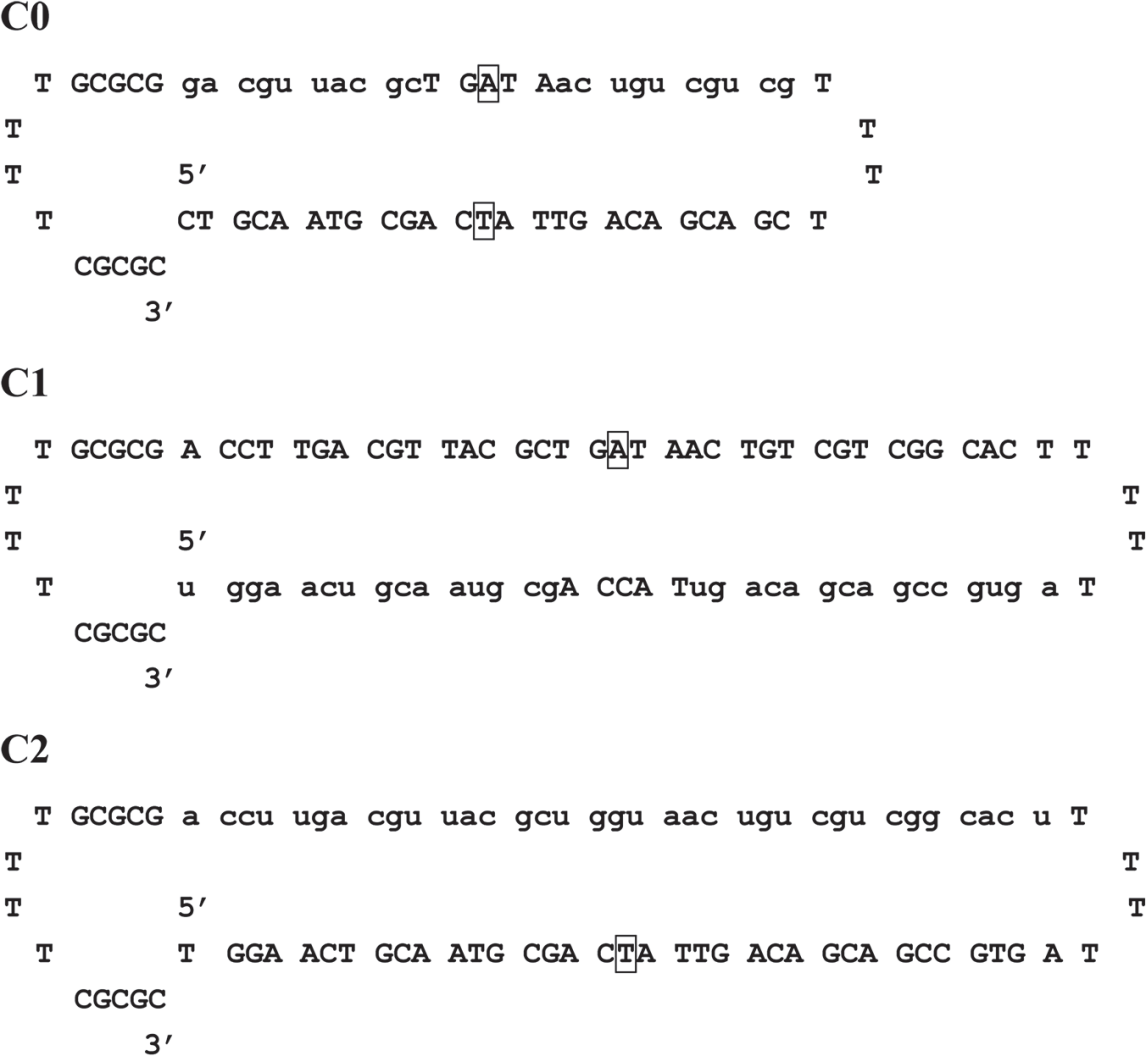
Chimeric RNA/DNA oligonucleotides (COs). Uppercase letters represent DNA residues, lowercase letters represent 2′-O-methyl-RNA residues, and the box indicates the nucleotide that should be introduced into the target sequence.

The plant material used in this study was rice Nipponbare (*Oryza sativa* L. ssp. *japonica*). Three COs were designed based on the sequence of the target *OsEPSPS* gene of Nipponbare (Locus number LOC_Os06g04280 in Rice Genome Annotation Project) and synthesized by TaKaRa Biotechnology (Dalian, China). The related bases are as follows: TTG GGG AAC GCT GGA ACT GCA ATG CGA CCA (change to CTA, which encodes a Leu instead of a Pro) TTG ACA GCA GCC GTG ACT. C0 (Fig 1) was designed based on the classic structure of COs, which has been used in many studies in plants [20,22,23], while C1 and C2 (Fig 1) were designed according to Gamper et al., who found that some kinds of COs with altered structures (similar to C1 and C2) exhibited highly activity in correcting a mutant gene in human cell-free extract [28]. We also increased the amount of DNA and RNA residues from 68 to 88 in C1 and C2 for further increasing their activity.

**Fig 2:**
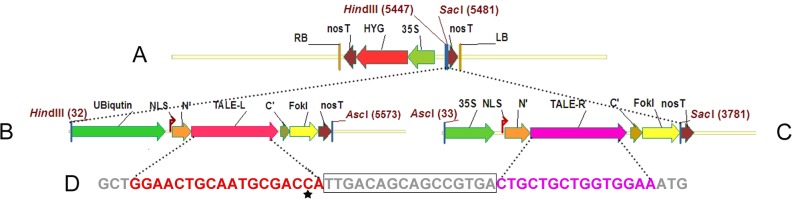
TALEN discrimination sequence and vector construction. (A) pCAMBIA1301M for plant transformation. (B) TALEN left-arm cassette L20. (C) TALEN right-arm cassette R16. (D) The discrimination sequence and target site in rice genome. The asterisk indicates the target base C^317^ and the box indicates the spacer sequence.

The plasmid pCAMBIA1301M that contains a hygromycin phosphotransferase gene (*hyg*) was used as the backbone for TALEN construction (Fig 2A). The left and right arm of sequence-specific TALEs (TALE-L and TALE-R) were designed and assembled with the Fast TALEN Assembly Kit (SIDANSAI) according to the manufacturer’s instructions. Both the left-arm vector pL20 and right-arm vector pR16 are containing Fok I DNA cleavage domain, Nos-T-terminus, and nuclear localization signals (NLSs). An ubiquitin promoter and a 35S promoter from the cauliflower mosaic virus are inserted into pL20 and pR16, respectively (Fig 2B and 2C). The above two TALEN expression cassettes were excised with *Hin*dIII/*Asc*I at pL20 and *Asc*I/*Sac*I at pR16 and moved into the *Hin*dIII/*Sac*I site of the plasmid pCAMBIA1301M, resulting in pCAMBIA1301M-TALEN. In order to avoid cleaving by TALEN again, the target base C^317^ was designed to be located on the TALE-L binding sequence so as to prevent its discrimination in the case of successful base substitution (Fig 2D).

The HRP was constructed by inserting a modified 898 bp-homologous fragment (Supplementary sequence 4) without promoter into a pMD18-T vector (TaKaRa) via TA cloning, resulting in pMD-EPSPM. The homologous fragment was amplified from the genomic DNA of Nipponbare with the primers EMS (5′-ATTATGCAATTTTGAGGCTCCT-3′) and EMR (5′-ACTGTGCAGTGTAAGGTCGAAT-3′). A single base substitution corresponding to the target site (C^317^-T) was introduced into the homologous fragment by overlapping PCR technology. The homologous fragment was 508 bp upstream and 389 bp downstream of the target base. The whole sequence of pMD-EPSPM was approximately 3.5 kb.

### Transformation Procedure and Growth Conditions

The embryogenic calli induced from the scutellum of mature seeds were subcultured on N6 medium [29] supplemented with 2 mg/L 2,4-dichlorophenoxyacetic acid, 2 g/L casamino acids, 3 g/L gelrite, and 30 g/L sucrose. The calli were placed on a plate in a circle that was 2.5 cm in diameter for delivery of the molecular constructs.

COs and plasmids were delivered to the calli by particle bombardment according to Okuzaki and Toriyama [23]. Ten μg donor molecules (COs or HRP) together with 10 μg pCAMBIA1301M-TALEN were co-precipitated with 100 μL 2.5 mM CaCl_2_ and 40 μL 0.1 M spermidine (Sigma) onto 6 mg 1.1-μm gold particles (Bio-Rad, Hercules, USA), and then resuspended in 120 μL 100% ethanol. For transformation of TALEN alone, 20 μg of pCAMBIA1301M-TALEN was used (Table 1). For COs together with selectable hygromycin resistance gene mediated GT, 10 μg COs together with 10 μg *hyg* containing pCAMBIA1300 plasmid were used for the transformation. An aliquot (10 μL) of the resuspended solution was used for one shoot and each plate of calli was shot twice for improving the transformation efficiency. A total of six plates of calli were bombarded with 12 shoots for each transformation. The bombardment was carried out at 1100 psi with a target distance of 9 cm, using a PDS-1000 He delivery system (Bio-Rad).

**Table 1:** Gene targeting by transformation of TALEN independently or co-transformation with donor molecules.

Treatment	Positive calli	Mutant	Mutant with C-T
**TALEN**	19	4	0
**TALEN + pMD-EPSPM**	30	5	0
**TALEN + C1**	25	3	1
**TALEN + C2**	31	5	0
**TOTAL**	105	17	1

Three days after bombardment, the calli were transferred onto N6 medium supplemented with 2 mg/L 2,4-dichlorophenoxyacetic acid, 2 g/L casamino acids, 3 g/L gelrite, 30 g/L sucrose, and 50 mg/L hygromycin for three cycles, each cycle last 14 days. The resulted resistant calli were selected and regenerated on N6 medium supplemented with 4 mg/L 6-benzylaminopurine, 1 mg/L α-naphthaleneacetic acid, 30 g/L sucrose, 5 g/L gelrite, and 25 mg/L hygromycin. The subsequent plantlets were transplanted in Yoshida’s culture solution [30] for one week and then transplanted in soil in greenhouse under standard growth conditions (12-h light at 30°C rotated with 12-h darkness at 22°C). The average of light intensity was 30000 lux.

### Molecular Analysis and Herbicide Test

Genomic DNA was extracted from the HYG-resistant calli or fresh leaves of the regenerated plants. The target sequences corresponding to *OsEPSPS* gene were amplified from the extracted genomic DNA using Prime STAR HS Taq polymerase (TaKaRa) and the primers of EF (5′- CAACTTTGGAGGTTTCGCACTG-3′) and ER (5′-TCGCTTGAGCTTGGCAGGAATA-3′). EF and ER were located outside the homologous sequence in the *OsEPSPS* gene thus also fit for amplifing the target sequences from the transformation with TALEN and HRP. Each callus or plant line was sampled twice for PCR amplification. Then, the resulted PCR products (1023 bp) were subjected to sequencing. If the amplicons were derived from heterozygous or chimeric mutants, the resulted sequencing maps were presented in polymorphism peaks in the target site. These PCR products were then inserted into the pMD18-T vector (TaKaRa) and transformed into *E*. *coli* by heat shock at 42°C for 1 min. To verify the detailed mutation sequences, at least 30 subsequent colonies in each bacterial transformation were randomly selected and subjected to sequencing. The amount and ratio of colonies contained different sequences would help to evaluate the variety and frequency of the mutations in each mutant.

In the co-transformation of TALEN and HRP, the homologous fragments in the HRP were designed to recombine with the target sequence, however, these homologous fragments and/or other fragments within the HRP might also inserted randomly into other sites of the rice genome via biolistic bombardment. To detect the possible random insertions, two pairs of primers which were located on the backbone of the vector pMD18-T were used: PMD18F (5′-TACCCGGGGATCCTCTAGAGA-3′)/PMD18R (5′-ATGCCTGCAGGTCGACGAT-3′) and M13F -47 (5′-CGCCAGGGTTTTCCCAGTCACGAC-3′)/M13R-48 (5′-AGCGGATAACAATTTCACACAGGA-3′); both the resulted products were approximately 1 kb. To detect the TALEN constructs that inserted into the rice genome, the primers located on either the TALEN construct (305/306) or the *hyg* gene (HF/HR) were used, since the TALEN construct was located on the same T-DNA as the *hyg* gene in the plasmid pCAMBIA1301M-TALEN. For HF (5′-GCTGTTATGCGGCCATTGTC-3′) and HR (5′-GACGTCTGTCGAGAAGTTTC-3′) the resulted product was 615 bp while for 305 (5′-CTCCCCTTCAGCTGGACAC-3′) and 306 (5′-AGCTGGGCCACGATTGAC-3′) the product was approximately 2 kb.

For herbicide test in plants, glyphosate solution Roundup (41.0% of isopropylamine glyphosate salt as active ingredient, Monsanto) was used at 300× dilution. The symptoms were scored and photographed 10 days after the treatment.

## Results

### GT by Co-transformation of TALEN and COs/HRP

**Fig 3:**
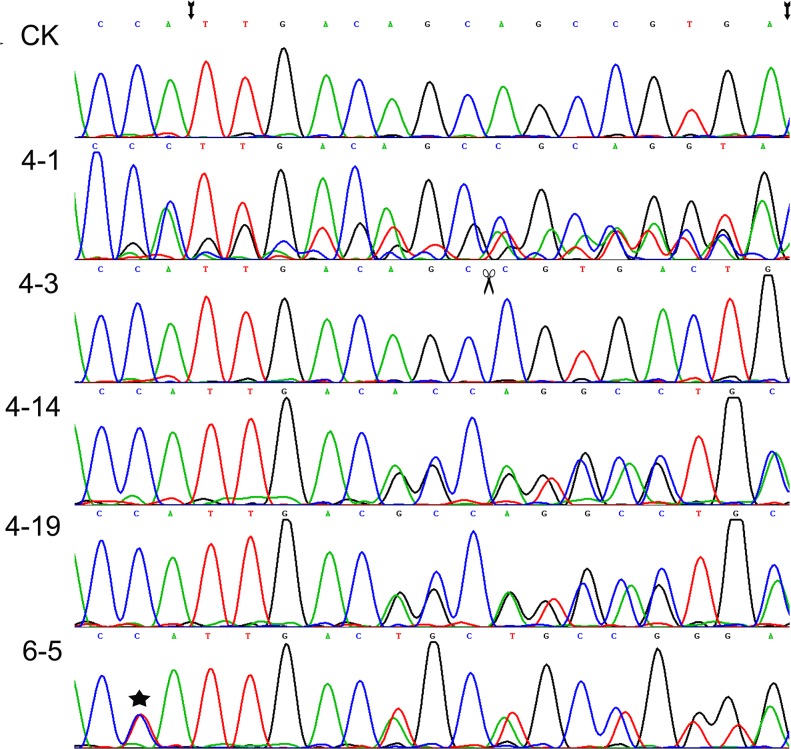
Sequencing maps of the PCR product of some calli lines in the T0 generation. 4–1 is a chimera, 4–3 is a homozygote, and 4–14, 4–19, and 6–5 are heterozygotes. The sequence between two arrows is the spacer cut by TALEN. The scissors indicate three-base deletions and the asterisk indicates the targeted base substitution C^317^-T.

To test the cleaving activity of the designed TALEN construct in rice, the pCAMBIA1301M-TALEN was first transformed independently into rice calli via biolistic bombardment. Four mutants with bases deletions were found after sequencing all of the 19 independent hygromycin-resistant calli lines on the target locus (Table 1 and Fig 3). The high mutation rate (21%) indicated that the TALEN construct had sufficient activity to introduce a DSB at the target site. Then, to test whether the DSB can promote base substitution, we bombarded the calli with the TALEN construct plus three donor molecules (HRP, C1 and C2, respectively), which were designed to replace the C^317^ by T in the genome. The resulting hygromycin-tolerant calli were selected and the target sequences were amplified and subjected to sequencing. Mutations were identified in 13 out of 86 independent transgenic calli (15.1%). Most of the mutants contained base deletions in the target sequence, but failed to substitute the target base C^317^ (Tables 1 and 2). Only one mutant derived from the co-transformation with TALEN and C1 involved a substitution of C^317^-T, accompanied by 12 bases deletion (Tables 1 and 2 and Fig 3).

**Table 2:** Variety of mutations induced by TALEN and donor molecules on the *OsEPSPS* target.

Transformant	Line	Sequence (5′-3′)	Genotype	Variation
**CK**		*GGAACTGCAATGCGAC* ***C*** *A*TTGACAGCAGCCGTGA*CTGCTGCTGGTGGAA*ATGCAACGTATG	Homozygote	WT
**TALEN**	4–1	GGAACTGCAATGCGACCATTGACAGCAGCCGTGACTGCTGCTGGTGGAAATGCAACGTATG	Chimera	WT
		GGAACTGCAATGCGACCATTGA—-CAGCCGTGACTGCTGCTGGTGGAAATGCAACGTATG		d_3_
		GGAACTGCAATGCGAC——————————GCTGCTGGTGGAAATGCAACGTATG		d_20_
		GGAACTGCAATGCGAC——————CCGTGACTGCTGCTGGTGGAAATGCAACGTATG		d_12_
	4–3	GGAACTGCAATGCGACCATTGACAGC—-CGTGACTGCTGCTGGTGGAAATGCAACGTATG	Homozygote	d_3_
		GGAACTGCAATGCGACCATTGACAGC—-CGTGACTGCTGCTGGTGGAAATGCAACGTATG		d_3_
	4–14	GGAACTGCAATGCGACCATTGACAGCAGCCGTGACTGCTGCTGGTGGAAATGCAACGTATG	Heterozygote	WT
		GGAACTGCAATGCGACCATTGAC——GCCGTGACTGCTGCTGGTGGAAATGCAACGTATG		d_4_
	4–19	GGAACTGCAATGCGACCATTGACAGCAGCCGTGACTGCTGCTGGTGGAAATGCAACGTATG	Heterozygote	WT
		GGAACTGCAATGCGACCATTGAC——GCCGTGACTGCTGCTGGTGGAAATGCAACGTATG		d_4_
**TALEN+pMD-EPSPM**	5–1	GGAACTGCAATGCGACCATTGACAGCAGCCGTGACTGCTGCTGGTGGAAATGCAACGTATG	Heterozygote	WT
		GGAACTGCAATGCGACCATTGAC——————TGCTGCTGGTGGAAATGCAACGTATG		d_12_
	5–7	GGAACTGCAATGCGACCATTGA—-CAGCCGTGACTGCTGCTGGTGGAAATGCAACGTATG	Homozygote	d_3_
		GGAACTGCAATGCGACCATTGA—-CAGCCGTGACTGCTGCTGGTGGAAATGCAACGTATG		d_3_
	5–12	GGAACTGCAATGCGACCATTGA—-CAGCCGTGACTGCTGCTGGTGGAAATGCAACGTATG	Homozygote	d_3_
		GGAACTGCAATGCGACCATTGA—-CAGCCGTGACTGCTGCTGGTGGAAATGCAACGTATG		d_3_
	5–16	GGAACTGCAATGCGACCATTGACAGCAGCCGTGACTGCTGCTGGTGGAAATGCAACGTATG	Heterozygote	WT
		GGAACTGCAATGCGACCATTGAC——GCCGTGACTGCTGCTGGTGGAAATGCAACGTATG		d_4_
	5–24	GGAACTGCAATGCGACCATTGACA——CCGTGACTGCTGCTGGTGGAAATGCAACGTATG	Chimera	d_4_
		GGAACTGCAATGCGACCATTGACA——-CGTGACTGCTGCTGGTGGAAATGCAACGTATG		d_5_
		GGAACTGCAATGCGACCATTGA—-CAGCCGTGACTGCTGCTGGTGGAAATGCAACGTATG		d_3_
**TALEN+C1**	6–5	GGAACTGCAATGCGACCATTGACAGCAGCCGTGACTGCTGCTGGTGGAAATGCAACGTATG	Heterozygote	WT
		GGAACTGCAATGCGAC**T**ATTGAC——————TGCTGCTGGTGGAAATGCAACGTATG		s_1_d_12_
	6–7	GGAACTGCAATGCGACCATTGACAGCAGCCGTGACTGCTGCTGGTGGAAATGCAACGTATG	Heterozygote	WT
		GGAACTGCAATGCGACCATTGA—-CAGCCGTGACTGCTGCTGGTGGAAATGCAACGTATG		d_3_
	6–22	GGAACTGCAATGCGACCATTGACAGCAGCCGTGACTGCTGCTGGTGGAAATGCAACGTATG	Chimera	WT
		GGAACTGCAATGCGAC————————————————————GTATG		d_40_
		GGAACTGCAATGCGACCATTGACA———-TGACTGCTGCTGGTGGAAATGCAACGTATG		d_7_
		GGAACTGCAATGCGACCATTGACAGC—-CGTGACTGCTGCTGGTGGAAATGCAACGTATG		d_3_
**TALEN+C2**	7–3	GGAACTGCAATGCGACCATTGACAGCAGCCGTGACTGCTGCTGGTGGAAATGCAACGTATG	Heterozygote	WT
		GGAACTGCAATGCGACCATTGACAG-AGCCGTGACTGCTGCTGGTGGAAATGCAACGTATG		d_1_
	7–14	GGAACTGCAATGCGACCATTGACAGCAGCCGTGACTGCTGCTGGTGGAAATGCAACGTATG	Heterozygote	WT
		GGAACTGCAATGCGACCATTGACAG-AGCCGTGACTGCTGCTGGTGGAAATGCAACGTATG		d_1_
	7–15	GGAACTGCAATGCGACCATTGACAGCAGCCGTGACTGCTGCTGGTGGAAATGCAACGTATG	Heterozygote	WT
		GGAACTGCAATGCGACCATT—————————————GAAATGCAACGTATG		d_26_
	7–24	GGAACTGCAATGCGACCATTGACAGCAGCCGTGACTGCTGCTGGTGGAAATGCAACGTATG	Heterozygote	WT
		GGAACTGCAATGCGACCATTGA—-CAGCCGTGACTGCTGCTGGTGGAAATGCAACGTATG		d_3_
	7–30	GGAACTGCAATGCGACCATTGACAGCAGCCGTGACTGCTGCTGGTGGAAATGCAACGTATG	Heterozygote	WT
		GGAACTGC———————————————TGCTGGTGGAAATGCAACGTATG		d_30_

Italics mark the TALEN-discrimination sequence. The letter in italic and bold (***C***) indicates the nucleotide that should be substituted by T. The letter in bold (T) indicates the nucleotide substitution of C-T. Each dashed line represents one deleted nucleotide. WT, wild-type sequence; d, base deletion; s, base substitution. The number after ‘d’ or ‘s’ represents the number of bases that were deleted or substituted.

Successful GT also requires free of random insertions of the donor DNAs. However, HRP (pMD-EPSPM) in the co-transformation events was not only failed to substitute the target base (Tables 1 and 2), but also randomly inserted its DNA fragments into the host genome via biolistic bombardment. Fourteen out of the 30 positive calli derived from the co-transformation of TALEN and HRP, including 5 mutant calli, were selected and subjected to PCR examination with the primers PMD18F/PMD18R and M13F-47/M13R-48. The results confirmed that 13 calli including the 5 mutants had both PCR products. These results indicated that the DNA fragments that derived from pMD-EPSPM were indeed inserted into rice genome randomly at a high frequency.

In our earlier experiments, each of the COs (C0, C1, and C2) was co-transformed into the rice calli with pCAMBIA1300. pCAMBIA1300 was acted as an associate screening marker for its contained *hyg* gene. A total of 452 independent hygromycin-resistant calli (92 from C1) were obtained, and 2266 regenerated plantlets were subjected to glyphosate test. Suspected glyphosate-resistant plantlets were further confirmed by sequencing. The result demonstrated that none of the plantlets contain either the predicted substitution of C^317^-T or any other mutations on the target locus (Table A in S1 File).

### Variety and Frequency of the Mutations Induced by TALEN on *OsEPSPS* Gene

After sequencing the target locus of all hygromycin-resistant calli derived from the transformation with TALEN, we found that the non-homologous end joining (NHEJ)-derived mutations resulted from TALEN activity could be classified into 3 types (Table 2). Some calli were found with both copies of the target sequence mutated with the same mutation, resulting in homozygotes (including lines 4–3, 5–7, and 5–12), or with only one copy mutated, resulting in heterozygotes, such as lines 4–14, 4–19, and 5–1. Feng et al. defined a chimera as an organism with at least 3 different sequences including wild type (WT) copy detected at the target site, thus a chimera must have different mutations in different cells [31]. In our study, 3 chimeric mutants were found among the 105 positive calli. Two of these lines, 4–1 and 6–22, both contained 3 kinds of mutated sequences except WT. The rest chimeric mutant (5–24) contained three kinds of mutated sequence without WT, indicating that partial cells in this callus with both copies of the target sequence mutated with different mutations (termed a bi-allelic mutation). The proportions of homozygotes, heterozygotes, and chimeras were 17.6%, 64.7%, and 17.6%, respectively. All mutations obtained in this study were short deletions, most of them were 3 bp; the shortest deletion was 1 bp while the longest was 40 bp (Table 2). Interestingly, the identical base deletions in different calli usually appeared in the same location of the target sequence. For example, up to 6 calli lines contained the same CAG deletion in the same location; 7–3 and 7–14 were the only two lines that contained a 1-bp deletion, and the deletion also appeared in the same location (Table 2).

### Mutants Containing Lethal Mutations Decreased in the Segregating Generation

**Fig 4:**
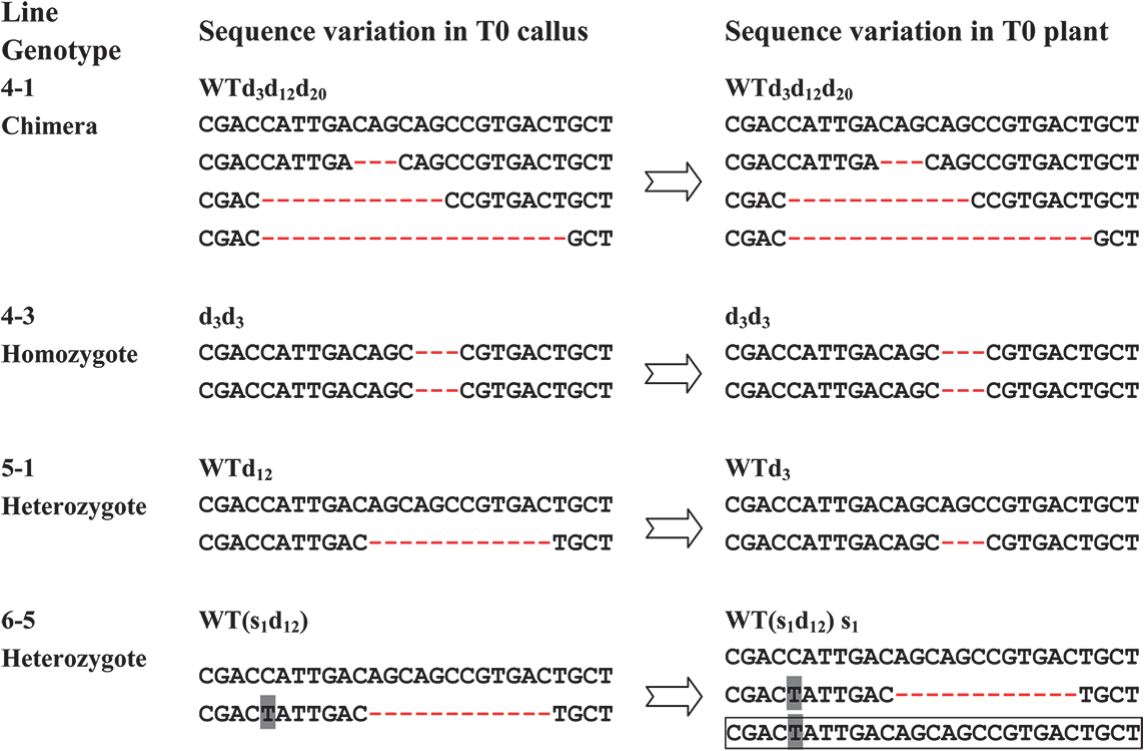
Mutations from the calli flow to the regenerated plants. The genotype and detailed sequence variation of some typical mutants from chimera, homozygote and heterozygote are presented. Each dashed line represents a deleted nucleotide. The nucleotide in gray represents substitution. WT, wild-type sequence; d, base deletion; s, base substitution. The number after ‘d’ or ‘s’ represents the number of bases that have been deleted or substituted. The box marks the variant sequence which might result from mismatch repair in *E*. *coli* during PCR product cloning.

The mutations that passed from the transgenic calli to the regenerated plants were examined first. Our data showed that although most of the 17 calli mutants were able to pass their mutations on to the regenerated plants, there were two exceptions. One in line 5–1 plants: a 3 bases-deletion was replaced the original 12 bases-deletion (Fig 4). It might due to that this line was actually a chimeric mutant. Another in line 6–5: a new mutation sequence with only the target base substitution (s_1_) presented in the regenerated plants (Fig 4). Since the s_1_ sequence only presented in one bacterial colony during the mutation verification, it might be either derived from the actual chimeric primary callus or as a result of mismatch repair in *E*. *coli* in the process of verification, as discussed later.

**Fig 5:**
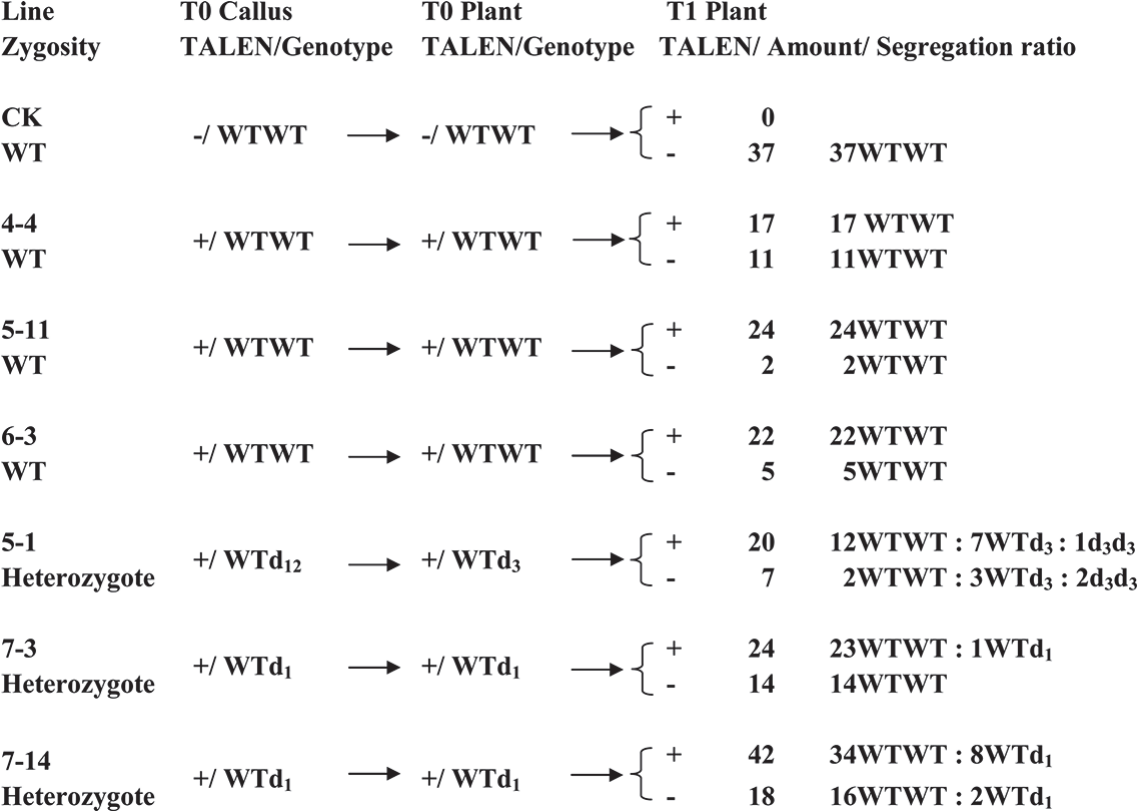
The segregation patterns of TALEN construct and the *OsEPSPS* genotype from T0 to T1 generation in some transgenic lines. T1 population are divided into two groups according to whether the TALEN construct exists (+) or not (–) in the genome. The number before genotype indicates the amount of T1 plants and the number after ‘d’ indicates the amount of bases that have been deleted. WT, wild-type sequence; d, base deletion.

Due to most of the regenerated plants, including the s_1_d_12_ mutant, were grown abnormally in T0 generation and few seeds had been harvested, we only selected 3 heterozygous mutants (5–1, 7–3, and 7–14) to explore the mutation’s inheritance from T0 plants to T1 populations. Their T1 plants were also subjected to glyphosate test. The genotype of line 5–1 with d_3_ mutation passed from the T0 plant to the T1 population with a ratio of 14WTWT:10WTd_3_:3d_3_d_3_ (Fig 5). A χ^2^ test showed that there was a significant difference from the expected 1:2:1 ratio if the mutations are inherited by the Mendelian pattern. The segregation patterns in lines 7–3 and 7–14 with d1 mutation were also inherited abnormally, with increase of the WT copy and decrease of the mutations. Moreover, no homozygous mutant recovered in T1 population in lines 7–3 and 7–14 (Fig 5).

**Fig 6:**
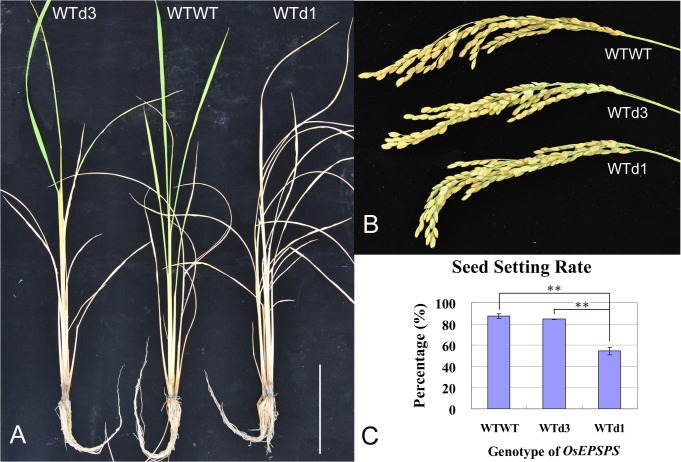
Phenotypic assessments of heterozygous *OsEPSPS* mutants with either one base deletion (WTd_1_) or three bases deletion (WTd_3_) and wild-type plant (WTWT) in T1 generation. A. Glyphosate test (Roundup 300×dilution). Heterozygous mutants are more sensitive to glyphosate; B. Spikelets; C. Seed-setting rate. Bar = 10 cm.

The rice *OsEPSPS* gene encodes a key enzyme in the synthesis of aromatic amino acids, loss of function in this gene results in a lack of essential amino acids, leading to plant death [26,32,33]. Line 5–1 contained d3 mutation, which led to deletion of one amino acid residue. Lines 7–3 and 7–14 both contained d1 mutation, which resulted in frameshift mutation. The phenotypic assessments showed that both heterozygous d1 and d3 plants segregated from lines 5–1, 7–3 and 7–14 in T1 generation became more sensitive to glyphosate (Fig 6A) than the WT plants. Moreover, the seed-setting rate of the heterozygous d1 plants in T1 generation was also decreased significantly from WT level of 87.3% to 54.4% (Fig 6B and 6C). These results therefore confirmed that the d1 and d3 mutants obtained via TALEN system are the real mutants. The decrease of seed-setting rate in heterozygous d1 plants and no segregation of d1 homozygotes in T1 generation indicated that the d1 mutation was lethal under homozygous condition. Nevertheless, we found that the mutations in T1 population were the same as their T0 generation. This suggested that the mutations caused by the TALEN system passed from the T0 to the T1 generation without any additional mutation, even when the TALEN construct was still present in some plants. Three lines (4–4, 5–11, and 6–3) that contained the TALEN construct but did not exhibit any mutation in the T0 generation still had the WT genotype in all of their T1 plants (Fig 5). This might due to the silencing of the TALEN construct in the host genome.

## Discussion

### The Possibility of GT with COs in Co-transformation Strategy

In the present study, 92 transgenic lines were generated in co-transformation of C1 with pCAMBIA1300, none of them presented modification on the target site. However, one mutant with C^317^-T substitution was obtained among only 25 transgenic lines derived from the co-transformation of C1 with TALEN system. In addition, none of the other 16 mutants derived from the transformations contained TALEN construct presented any kinds of base substitutions. These results indicated that the GT event with C1 was likely promoted by the DSB induced by TALEN, although accompanied by 12 base deletions due to the NHEJ effect. However, the only one target base substitution event might also directly derived from the NHEJ effect, or induced directly by C1 but irrelevant with the DSB.

It was well demonstrated that the HR with either dsDNA or ssDNA could be enhanced by an induced DSB [10,11,14,25]. However, whether the mismatch repair effect between COs and its target sequence can be promoted by DSB is still unclear. Nevertheless, the design of COs was prompted by the discovery that RNA/DNA hybrids were more active in homologous pairing reactions than corresponding DNA duplexes and the hairpin caps at the ends of hybrid molecules were no impediment to pairing [15,16]. As a result, GT process with COs in the stage of homologous pairing that occurs before the mismatch repair could be also promoted by an induced DSB. Since pairing would appear to be the rate-limiting step during the GT process, the overall frequency of GT should be elevated if the number of pairing events is elevated [16,34].

As GT donor, the amount of COs is dozens of times higher than that of HRP, due to their low molecular weight (generally less than 100 nt). Therefore, it is possible to introduce large amounts of these small donors to pass through the cell wall by biolistic bombardment. COs are also protected from exonucleolytic degradation by capping both ends. The 2'-O-methyl modification of ribose of the RNA further added protection against cleavage by ribonuclease activities. Therefore, the half-life in the cell and in serum is substantially longer than similar duplex molecules [16]. More over, COs are “clean” donors that will not insert into any part of the host genome. As a result, COs are theoretically reasonable donors in GT process by co-transformation with SSNs. However, the actual effect is still needed to be more rigorous tested.

### The Characteristic of Mutations Induced by TALEN on the *OsEPSPS* Gene

Zhang et al. found that all the detected CRISPR/Cas9-induced *OsEPSPS* mutations were 1 bp, most of them presented in base insertion [33]. However, only short deletions were obtained in the present study and most of them were 3–4 bases. This may be caused by the intrinsic function differences between these two nucleases. As most of the modifications were deletions, in calculating the TALEN-induced mutation patterns reported in previous studies, Kim et al. speculated that the reason for this might be associated with the comparatively large spacer region provided between the two TALEN binding sites [35].

The repeated recovery of SSNs-derived modifications has been reported in maize [36], zebrafish [37], rat [38], and barley [39]. Tesson et al (2011) suggested the possibility that microhomology-mediated reclosure of DSBs could explain the phenomenon [38]. On the other hand, Gurushidze et al. (2014) found that two primary mutants carried an identical 36 nucleotide deletion, which was recovered from the same experiment, and thus speculated that the two individuals were in fact derived from a single mutagenesis event [39]. In the present study, however, some identical deletions with certain length of nucleotides were repeatedly presented in different transformation events. This phenomenon might be due to those identical deletions did not lead to frameshift mutations or loss of key residues and thus were not lethal to the recipient cell. But in contrast some deletions excluded in the above category were either led to frameshift mutations or loss of key residues and thus rendered the cell unviable due to *OsEPSPS* is an indispensable endogenous gene for rice survival, so that we had no opportunity to detect those lethal types of mutations. Therefore, we had the reason to believe that the actual activity of our constructed TALEN system was much higher than what could be seen in the results.

### Variations in the Mutation Flow

Combining this study with previous studies, the possible variations of the mutations in passing to the next generation can be divided into several situations.

One situation is that if no SSNs construct insertion occurred or the inserted SSNs construct is incomplete or silenced, generally no mutations could be found in any generation. These have been demonstrated in *Arabidopsis* [40] and rice [33]. In the present study, we examined the T1 populations of three lines (4–4, 5–11, and 6–3) that contained the TALEN construct but showed no mutation in their T0 generation, and the results showed that there were still no mutations in any of the T1 plants, possibly due to the incomplete or silencing of the inserted TALEN constructs. However, even in these cases the transient expression of SSNs may also function in the host genome. Gurushidze et al. found that 4 induced mutants possessed only a single stably integrated TALEN unit, as confirmed by DNA gel blot analysis [39]. The creation of DSBs requires FokI dimerization, so the assumption was that the non-integrated TALEN unit must have only been transiently expressed in the recipient cells from which these mutants were derived. A similar non-integration of a TALEN construct was also observed in experiments conducted using tobacco protoplast-derived calli [3].

Another situation is that the mutated sequences are usually passed stably to the next generation without new mutations or reversions. Zhu and his colleagues found that the gene mutations induced by CRISPR/Cas9 system in either *Arabidopsis* or rice were passed to the next generation without any detectable new mutation or reversion [31,33]. Gurushidze et al. proposed that the early disruption of the TALEN binding sites in barley cells avoids repeated binding of the TALENs, thereby ensuring that no further diversification of mutations will be induced in subsequent daughter cells [39]. In our study, we also demonstrated that all of the mutations examined in the T1 population were the same as that of the T0 plants. These results indicate that both TALEN and the CRISPR/Cas9 system are highly specific in discriminating their target sequence.

The third situation is that the continued activity of the SSNs is usually occurred on the WT copy in the chimeric or heterozygous mutants. The mutation itself has already demonstrated the activity of the inserted SSNs in these mutants, thus the intact WT copy is still an ideal target for the continued activity of these nucleases. Zhang et al. showed that the WT copy of the target gene in heterozygous and chimeric rice plants could continue to mutate either in the T0 or T1 generation [33]. Feng et al. also demonstrated that T2 chimeras of *Arabidopsis* exhibited complex patterns in transmitting mutations to the T3 generation [31]. Gurushidze et al. found that multiple targeted alleles were observed in some progenies that had not been detected in the T0 primary mutant [39]. The TALEN units might have kept binding the target sites repeatedly producing new mutant alleles until at least one of the target sites had been destroyed, that rendered the target region inaccessible to the TALE nucleases.

The fourth situation, there is a decrease in the amount of mutants that containing lethal mutations. TALEN system has been successfully applied in target gene editing in many plants, such as *Arabidopsis* [40], rice [2,5], barley [39], soybean [41], wheat [14], *Brachypodium* [5], and tobacco [3]. Some of the above studies have demonstrated that the induced mutations were transmitted to next generation in Mendelian fashion [14,40,41]. However, in the present study we found that the segregation patterns of some mutations in T1 population did not fit the Mendelian ratio. Some mutations may be less detrimental to the function of the gene, so that the plant that carries these mutations has a better chance to survive, but other mutant cells that were lethal, or caused growth disadvantages, may be removed during propagation and segregation. The target locus *OsEPSPS* is a key gene involved in the aromatic amino acid synthesis pathway, mutations of both copies of the gene may render the plant unviable. As a result, Zhang et al. found that no T1 *OsEPSPS* homozygous or bi-allelic mutants were recovered after editing by CRISPR/Cas9 system [33]. In this study, we also found that the heterozygous mutants 5–1, 7–3, and 7–14 did not inherit following Mendel’s law, with the mutants decreased and WT plants increased in T1 generation. Moreover, no homozygous mutants were recovered in the T1 populations derived from lines 7–3 and 7–14 containing d_1_ mutation.

The fifth situation is in the chimeric type of positive calli or plants, where the wild and/or the mutated target sequences appear in different callus samples or tissues or organs. As a consequence not all of these individual parts of chimeras equally contribute to the germ line, thus resulting in mutation variations when verifying the target sequence in different callus samples or tissues or organs or even in different generations.

The last situation is in the process of verifying target mutations, where the PCR reactions were carried out using the mix-DNA-templates that derived from heterozygous or chimeric mutants and hence likely to generate the heteroduplex DNAs. Therefore, the transformation of these heteroduplex DNAs into *E*. *coli* may generate new sequence variation by the mismatch repair system existing in *E*. *coli*, and present in the individual clones. In the detailed sequence identification of the plant regenerated from the callus line 6–5, the mutant sequence s_1_ only appeared in one bacterial colony, while both WT and s_1_d_12_ sequences arose in more than 10 of the 30 selected bacterial colonies. However, in the repeated identification of the same T0 plant regenerated from the callus line 6–5, the s_1_ sequence was never emerged again. Based on this, we speculated that this mutation sequence might be an artifact derived from the verification process. Of course, as the fifth situation discussed above, it might be also derived from an actual chimeric primary callus.

## Supporting Information

S1 File Results of gene targeting by co-transformation with COs and a selected marker and the sequence information related to this research. Table A in S1 File: Results of gene targeting by co-transformation with COs and a selected marker (*hyg* gene) containing plasmid pCAMBIA1300. Sequences Information in S1 File: The sequence of pCAMBIA1301M; The discrimination sequence and coding sequence of TALE-L and TALE-R; The sequence of pL20 and pR16; The 898 bp-homologous fragment contained in the HRP (pMD-EPSPM).
(DOC)
