# Supplementary material for: Gene Editing by Co-Transformation of TALEN and Chimeric RNA/DNA Oligonucleotides on the Rice OsEPSPS Gene and the Inheritance of Mutations
Source: PLoS One. 2015 Apr 9;10(4):e0122755. doi: 10.1371/journal.pone.0122755 (PMC4391873; doi:10.1371/journal.pone.0122755)
Supplement: S1 File — Table A in S1 File: Results of gene targeting by co-transformation with COs and a selected marker (hyg gene) containing plasmid pCAMBIA1300. Sequences Information in S1 File: The sequence of pCAMBIA1301M; The discrimination sequence and coding sequence of TALE-L and TALE-R; The sequence of pL20 and pR16; The 898 bp-homologous fragment contained in the HRP (pMD-EPSPM). (DOC) [file pone.0122755.s001.doc]

**Supporting Information**

**Gene Editing by Co-transformation of TALEN and Chimeric RNA/DNA Oligonucleotides on the Rice *OsEPSPS* Gene and the Inheritance of Mutations**

Mugui Wang, Yujun Liu, Cuicui Zhang, Jianping Liu, Xin Liu, Liangchao Wang,

Wenyi Wang, Hao Chen, Chuchu Wei, Xiufen Ye, Xinyuan Li, Jumin Tu*

Institute of Crop Sciences, College of Agriculture and Biotechnology, Zhejiang University, Hangzhou Zhejiang Province, China

*Corresponding author

E-mail: jtu@zju.edu.cn

**Keywords**

Gene editing; Biolistic co-transformation; TALEN; Chimeric RNA/DNA oligonucleotides; DNA double strands break; *OsEPSPS*

**Table A. Results of gene targeting by co-transformation with COs and a selected marker (*hyg* gene) containing plasmid pCAMBIA1300.**

| **Treatment** | **Positive calli** | **Seedlings treated with glyphosate** | **Seedlings resistant to glyphosate** | **Seedlings selected for sequencing** | **Mutant** | **Mutant with C-T** |
| --- | --- | --- | --- | --- | --- | --- |
|
| **pCAMBIA1300 + C0** | 284 | 1422 | 0 | 44 | 0 | 0 |
| **pCAMBIA1300 + C1** | 92 | 458 | 0 | 22 | 0 | 0 |
| **pCAMBIA1300 + C2** | 76 | 386 | 0 | 19 | 0 | 0 |
| **TOTAL** | 452 | 2266 | 0 | 85 | 0 | 0 |

**Sequences Information**

**The sequence of pCAMBIA1301M** (LB-nosT-35S-HYG-nosT-RB)

CTATTTCTTTGCCCTCGGACGAGTGCTGGGGCGTCGGTTTCCACTATCGGCGAGTACTTCTACACAGCCATCGGTCCAGACGGCCGCGCTTCTGCGGGCGATTTGTGTACGCCCGACAGTCCCGGCTCCGGATCGGACGATTGCGTCGCATCGACCCTGCGCCCAAGCTGCATCATCGAAATTGCCGTCAACCAAGCTCTGATAGAGTTGGTCAAGACCAATGCGGAGCATATACGCCCGGAGTCGTGGCGATCCTGCAAGCTCCGGATGCCTCCGCTCGAAGTAGCGCGTCTGCTGCTCCATACAAGCCAACCACGGCCTCCAGAAGAAGATGTTGGCGACCTCGTATTGGGAATCCCCGAACATCGCCTCGCTCCAGTCAATGACCGCTGTTATGCGGCCATTGTCCGTCAGGACATTGTTGGAGCCGAAATCCGCGTGCACGAGGTGCCGGACTTCGGGGCAGTCCTCGGCCCAAAGCATCAGCTCATCGAGAGCCTGCGCGACGGACGCACTGACGGTGTCGTCCATCACAGTTTGCCAGTGATACACATGGGGATCAGCAATCGCGCATATGAAATCACGCCATGTAGTGTATTGACCGATTCCTTGCGGTCCGAATGGGCCGAACCCGCTCGTCTGGCTAAGATCGGCCGCAGCGATCGCATCCATAGCCTCCGCGACCGGTTGTAGAACAGCGGGCAGTTCGGTTTCAGGCAGGTCTTGCAACGTGACACCCTGTGAACGGCGGGAGATGCAATAGGTCAGGCTCTCGCTAAACTCCCCAATGTCAAGCACTTCCGGAATCGGGAGCGCGGCCGATGCAAAGTGCCGATAAACATAACGATCTTTGTAGAAACCATCGGCGCAGCTATTTACCCGCAGGACATATCCACGCCCTCCTACATCGAAGCTGAAAGCACGAGATTCTTCGCCCTCCGAGAGCTGCATCAGGTCGGAGACGCTGTCGAACTTTTCGATCAGAAACTTCTCGACAGACGTCGCGGTGAGTTCAGGCTTTTTCATAGATCCAGTCCCCCGTGTTCTCTCCAAATGAAATGAACTTCCTTATATAGAGGAAGGGTCTTGCGAAGGATAGTGGGATTGTGCGTCATCCCTTACGTCAGTGGAGATATCACATCAATCCACTTGCTTTGAAGACGTGGTTGGAACGTCTTCTTTTTCCACGATGCTCCTCGTGGGTGGGGGTCCATCTTTGGGACCACTGTCGGCAGAGGCATCTTCAACGATGGCCTTTCCTTTATCGCAATGATGGCATTTGTAGGAGCCACCTTCCTTTTCCACTATCTTCACAATAAAGTGACAGATAGCTGGGCAATGGAATCCGAGGAGGTTTCCGGATATTACCCTTTGTTGAAAAGTCTCAATTGCCCTTTGGTCTTCTGAGACTGTATCTTTGATATTTTTGGAGTAGACAAGTGTGTCGTGCTCCACCATGTTGACGAAGATTTTCTTCTTGTCATTGAGTCGTAAGAGACTCTGTATGAACTGTTCGCCAGTCTTTACGGCGAGTTCTGTTAGGTCCTCTATTTGAATCTTTGACTCCATGAAGCTAAACTGAAGGCGGGAAACGACAATCTGATCCAAGCTCAAGCTGCTCTAGCATTCGCCATTCAGGCTGTGCAACTGTTGGGAAGGGCGATCGGTGCGGGCCTCTTCGCTATTACGCCAGCTGGCGAAAGGGGGATGTGCTGCAAGGCGATTAAGTTGGGTAACGCCAGGGTTTTCCCAGTCACGACGTTGTAAAACGACGGCCAGTGCGAAGCTTCTGCAGGTCGACGAATTCACTAGTGAGCTCTCGACAAGCTCGAGTTTCTCCATAATAATGTGTGAGTAGTTCCCAGATAAGGGAATTAGGGTTCCTATAGGGTTTCGCTCATGTGTTGAGCATATAAGAAACCCTTAGTATGTATTTGTATTTGTAAAATACTTCTATCAATAAAATTTCTAATTCCTAAAACCAAAATCCAGTACTAAAATCCAGATCCCCCGAATTAATTCGGCGTTAATTCAGTACATTAAAAACGTCCGCAATGTGTTATTAAGTTGTCTAAGCGTCAATTTGTTTACACCACAATATATCCTGCCACCAGCCAGCCAACAGCTCCCCGACCGGCAGCTCGGCACAAAATCACCACTCGATACAGGCAGCCCATCAGTCCGGGACGGCGTCAGCGGGAGAGCCGTTGTAAGGCGGCAGACTTTGCTCATGTTACCGATGCTATTCGGAAGAACGGCAACTAAGCTGCCGGGTTTGAAACACGGATGATCTCGCGGAGGGTAGCATGTTGATTGTAACGATGACAGAGCGTTGCTGCCTGTGATCACCGCGGTTTCAAAATCGGCTCCGTCGATACTATGTTATACGCCAACTTTGAAAACAACTTTGAAAAAGCTGTTTTCTGGTATTTAAGGTTTTAGAATGCAAGGAACAGTGAATTGGAGTTCGTCTTGTTATAATTAGCTTCTTGGGGTATCTTTAAATACTGTAGAAAAGAGGAAGGAAATAATAAATGGCTAAAATGAGAATATCACCGGAATTGAAAAAACTGATCGAAAAATACCGCTGCGTAAAAGATACGGAAGGAATGTCTCCTGCTAAGGTATATAAGCTGGTGGGAGAAAATGAAAACCTATATTTAAAAATGACGGACAGCCGGTATAAAGGGACCACCTATGATGTGGAACGGGAAAAGGACATGATGCTATGGCTGGAAGGAAAGCTGCCTGTTCCAAAGGTCCTGCACTTTGAACGGCATGATGGCTGGAGCAATCTGCTCATGAGTGAGGCCGATGGCGTCCTTTGCTCGGAAGAGTATGAAGATGAACAAAGCCCTGAAAAGATTATCGAGCTGTATGCGGAGTGCATCAGGCTCTTTCACTCCATCGACATATCGGATTGTCCCTATACGAATAGCTTAGACAGCCGCTTAGCCGAATTGGATTACTTACTGAATAACGATCTGGCCGATGTGGATTGCGAAAACTGGGAAGAAGACACTCCATTTAAAGATCCGCGCGAGCTGTATGATTTTTTAAAGACGGAAAAGCCCGAAGAGGAACTTGTCTTTTCCCACGGCGACCTGGGAGACAGCAACATCTTTGTGAAAGATGGCAAAGTAAGTGGCTTTATTGATCTTGGGAGAAGCGGCAGGGCGGACAAGTGGTATGACATTGCCTTCTGCGTCCGGTCGATCAGGGAGGATATCGGGGAAGAACAGTATGTCGAGCTATTTTTTGACTTACTGGGGATCAAGCCTGATTGGGAGAAAATAAAATATTATATTTTACTGGATGAATTGTTTTAGTACCTAGAATGCATGACCAAAATCCCTTAACGTGAGTTTTCGTTCCACTGAGCGTCAGACCCCGTAGAAAAGATCAAAGGATCTTCTTGAGATCCTTTTTTTCTGCGCGTAATCTGCTGCTTGCAAACAAAAAAACCACCGCTACCAGCGGTGGTTTGTTTGCCGGATCAAGAGCTACCAACTCTTTTTCCGAAGGTAACTGGCTTCAGCAGAGCGCAGATACCAAATACTGTCCTTCTAGTGTAGCCGTAGTTAGGCCACCACTTCAAGAACTCTGTAGCACCGCCTACATACCTCGCTCTGCTAATCCTGTTACCAGTGGCTGCTGCCAGTGGCGATAAGTCGTGTCTTACCGGGTTGGACTCAAGACGATAGTTACCGGATAAGGCGCAGCGGTCGGGCTGAACGGGGGGTTCGTGCACACAGCCCAGCTTGGAGCGAACGACCTACACCGAACTGAGATACCTACAGCGTGAGCTATGAGAAAGCGCCACGCTTCCCGAAGGGAGAAAGGCGGACAGGTATCCGGTAAGCGGCAGGGTCGGAACAGGAGAGCGCACGAGGGAGCTTCCAGGGGGAAACGCCTGGTATCTTTATAGTCCTGTCGGGTTTCGCCACCTCTGACTTGAGCGTCGATTTTTGTGATGCTCGTCAGGGGGGCGGAGCCTATGGAAAAACGCCAGCAACGCGGCCTTTTTACGGTTCCTGGCCTTTTGCTGGCCTTTTGCTCACATGTTCTTTCCTGCGTTATCCCCTGATTCTGTGGATAACCGTATTACCGCCTTTGAGTGAGCTGATACCGCTCGCCGCAGCCGAACGACCGAGCGCAGCGAGTCAGTGAGCGAGGAAGCGGAAGAGCGCCTGATGCGGTATTTTCTCCTTACGCATCTGTGCGGTATTTCACACCGCATATGGTGCACTCTCAGTACAATCTGCTCTGATGCCGCATAGTTAAGCCAGTATACACTCCGCTATCGCTACGTGACTGGGTCATGGCTGCGCCCCGACACCCGCCAACACCCGCTGACGCGCCCTGACGGGCTTGTCTGCTCCCGGCATCCGCTTACAGACAAGCTGTGACCGTCTCCGGGAGCTGCATGTGTCAGAGGTTTTCACCGTCATCACCGAAACGCGCGAGGCAGGGTGCCTTGATGTGGGCGCCGGCGGTCGAGTGGCGACGGCGCGGCTTGTCCGCGCCCTGGTAGATTGCCTGGCCGTAGGCCAGCCATTTTTGAGCGGCCAGCGGCCGCGATAGGCCGACGCGAAGCGGCGGGGCGTAGGGAGCGCAGCGACCGAAGGGTAGGCGCTTTTTGCAGCTCTTCGGCTGTGCGCTGGCCAGACAGTTATGCACAGGCCAGGCGGGTTTTAAGAGTTTTAATAAGTTTTAAAGAGTTTTAGGCGGAAAAATCGCCTTTTTTCTCTTTTATATCAGTCACTTACATGTGTGACCGGTTCCCAATGTACGGCTTTGGGTTCCCAATGTACGGGTTCCGGTTCCCAATGTACGGCTTTGGGTTCCCAATGTACGTGCTATCCACAGGAAAGAGACCTTTTCGACCTTTTTCCCCTGCTAGGGCAATTTGCCCTAGCATCTGCTCCGTACATTAGGAACCGGCGGATGCTTCGCCCTCGATCAGGTTGCGGTAGCGCATGACTAGGATCGGGCCAGCCTGCCCCGCCTCCTCCTTCAAATCGTACTCCGGCAGGTCATTTGACCCGATCAGCTTGCGCACGGTGAAACAGAACTTCTTGAACTCTCCGGCGCTGCCACTGCGTTCGTAGATCGTCTTGAACAACCATCTGGCTTCTGCCTTGCCTGCGGCGCGGCGTGCCAGGCGGTAGAGAAAACGGCCGATGCCGGGATCGATCAAAAAGTAATCGGGGTGAACCGTCAGCACGTCCGGGTTCTTGCCTTCTGTGATCTCGCGGTACATCCAATCAGCTAGCTCGATCTCGATGTACTCCGGCCGCCCGGTTTCGCTCTTTACGATCTTGTAGCGGCTAATCAAGGCTTCACCCTCGGATACCGTCACCAGGCGGCCGTTCTTGGCCTTCTTCGTACGCTGCATGGCAACGTGCGTGGTGTTTAACCGAATGCAGGTTTCTACCAGGTCGTCTTTCTGCTTTCCGCCATCGGCTCGCCGGCAGAACTTGAGTACGTCCGCAACGTGTGGACGGAACACGCGGCCGGGCTTGTCTCCCTTCCCTTCCCGGTATCGGTTCATGGATTCGGTTAGATGGGAAACCGCCATCAGTACCAGGTCGTAATCCCACACACTGGCCATGCCGGCCGGCCCTGCGGAAACCTCTACGTGCCCGTCTGGAAGCTCGTAGCGGATCACCTCGCCAGCTCGTCGGTCACGCTTCGACAGACGGAAAACGGCCACGTCCATGATGCTGCGACTATCGCGGGTGCCCACGTCATAGAGCATCGGAACGAAAAAATCTGGTTGCTCGTCGCCCTTGGGCGGCTTCCTAATCGACGGCGCACCGGCTGCCGGCGGTTGCCGGGATTCTTTGCGGATTCGATCAGCGGCCGCTTGCCACGATTCACCGGGGCGTGCTTCTGCCTCGATGCGTTGCCGCTGGGCGGCCTGCGCGGCCTTCAACTTCTCCACCAGGTCATCACCCAGCGCCGCGCCGATTTGTACCGGGCCGGATGGTTTGCGACCGTCACGCCGATTCCTCGGGCTTGGGGGTTCCAGTGCCATTGCAGGGCCGGCAGACAACCCAGCCGCTTACGCCTGGCCAACCGCCCGTTCCTCCACACATGGGGCATTCCACGGCGTCGGTGCCTGGTTGTTCTTGATTTTCCATGCCGCCTCCTTTAGCCGCTAAAATTCATCTACTCATTTATTCATTTGCTCATTTACTCTGGTAGCTGCGCGATGTATTCAGATAGCAGCTCGGTAATGGTCTTGCCTTGGCGTACCGCGTACATCTTCAGCTTGGTGTGATCCTCCGCCGGCAACTGAAAGTTGACCCGCTTCATGGCTGGCGTGTCTGCCAGGCTGGCCAACGTTGCAGCCTTGCTGCTGCGTGCGCTCGGACGGCCGGCACTTAGCGTGTTTGTGCTTTTGCTCATTTTCTCTTTACCTCATTAACTCAAATGAGTTTTGATTTAATTTCAGCGGCCAGCGCCTGGACCTCGCGGGCAGCGTCGCCCTCGGGTTCTGATTCAAGAACGGTTGTGCCGGCGGCGGCAGTGCCTGGGTAGCTCACGCGCTGCGTGATACGGGACTCAAGAATGGGCAGCTCGTACCCGGCCAGCGCCTCGGCAACCTCACCGCCGATGCGCGTGCCTTTGATCGCCCGCGACACGACAAAGGCCGCTTGTAGCCTTCCATCCGTGACCTCAATGCGCTGCTTAACCAGCTCCACCAGGTCGGCGGTGGCCCATATGTCGTAAGGGCTTGGCTGCACCGGAATCAGCACGAAGTCGGCTGCCTTGATCGCGGACACAGCCAAGTCCGCCGCCTGGGGCGCTCCGTCGATCACTACGAAGTCGCGCCGGCCGATGGCCTTCACGTCGCGGTCAATCGTCGGGCGGTCGATGCCGACAACGGTTAGCGGTTGATCTTCCCGCACGGCCGCCCAATCGCGGGCACTGCCCTGGGGATCGGAATCGACTAACAGAACATCGGCCCCGGCGAGTTGCAGGGCGCGGGCTAGATGGGTTGCGATGGTCGTCTTGCCTGACCCGCCTTTCTGGTTAAGTACAGCGATAACCTTCATGCGTTCCCCTTGCGTATTTGTTTATTTACTCATCGCATCATATACGCAGCGACCGCATGACGCAAGCTGTTTTACTCAAATACACATCACCTTTTTAGACGGCGGCGCTCGGTTTCTTCAGCGGCCAAGCTGGCCGGCCAGGCCGCCAGCTTGGCATCAGACAAACCGGCCAGGATTTCATGCAGCCGCACGGTTGAGACGTGCGCGGGCGGCTCGAACACGTACCCGGCCGCGATCATCTCCGCCTCGATCTCTTCGGTAATGAAAAACGGTTCGTCCTGGCCGTCCTGGTGCGGTTTCATGCTTGTTCCTCTTGGCGTTCATTCTCGGCGGCCGCCAGGGCGTCGGCCTCGGTCAATGCGTCCTCACGGAAGGCACCGCGCCGCCTGGCCTCGGTGGGCGTCACTTCCTCGCTGCGCTCAAGTGCGCGGTACAGGGTCGAGCGATGCACGCCAAGCAGTGCAGCCGCCTCTTTCACGGTGCGGCCTTCCTGGTCGATCAGCTCGCGGGCGTGCGCGATCTGTGCCGGGGTGAGGGTAGGGCGGGGGCCAAACTTCACGCCTCGGGCCTTGGCGGCCTCGCGCCCGCTCCGGGTGCGGTCGATGATTAGGGAACGCTCGAACTCGGCAATGCCGGCGAACACGGTCAACACCATGCGGCCGGCCGGCGTGGTGGTGTCGGCCCACGGCTCTGCCAGGCTACGCAGGCCCGCGCCGGCCTCCTGGATGCGCTCGGCAATGTCCAGTAGGTCGCGGGTGCTGCGGGCCAGGCGGTCTAGCCTGGTCACTGTCACAACGTCGCCAGGGCGTAGGTGGTCAAGCATCCTGGCCAGCTCCGGGCGGTCGCGCCTGGTGCCGGTGATCTTCTCGGAAAACAGCTTGGTGCAGCCGGCCGCGTGCAGTTCGGCCCGTTGGTTGGTCAAGTCCTGGTCGTCGGTGCTGACGCGGGCATAGCCCAGCAGGCCAGCGGCGGCGCTCTTGTTCATGGCGTAATGTCTCCGGTTCTAGTCGCAAGTATTCTACTTTATGCGACTAAAACACGCGACAAGAAAACGCCAGGAAAAGGGCAGGGCGGCAGCCTGTCGCGTAACTTAGGACTTGTGCGACATGTCGTTTTCAGAAGACGGCTGCACTGAACGTCAGAAGCCGACTGCACTATAGCAGCGGAGGGGTTGGATCAAAGTACTTTGATCCCGAGGGGAACCCTGTGGTTGGCATGCACATACAAATGGACGAACGGATAAACCTTTTCACGCCCTTTTAAATATCCGTTATTCTAATAAACGCTCTTTTCTCTTAGGTTTACCCGCCAATATATCCTGTCAAACACTGATAGTTTAATTCCCGATCTAGTAACATAGATGACACCGCGCGCGATAATTTATCCTAGTTTGCGCGCTATATTTTGTTTTCTATCGCGTATTAAATGTATAATTGCGGGACTCTAATCATAAAAACCCATCTCATAAATAACGTCATGCATTACATGTTAATTATTACATGCTTAACGTAATTCAACAGAAATTATATGATAATCATCGCAAGACCGGCAACAGGATTCAATCTTAAGAAACTTTATTGCCAAATGTTTGAACGATC

**The discrimination sequence and coding sequence of TALE-L and TALE-R**

**TALE-L: GGAACTGCAATGCGACCA**

CTCACTCCGGAACAAGTGGTCGCAATCGCGAGCAATAACGGCGGAAAACAGGCTTTGGAAACGGTGCAGAGGCTCCTTCCAGTGCTGTGCCAAGCGCACGGTCTCACTCCGGAACAAGTGGTCGCAATCGCGAGCAATAACGGCGGAAAACAGGCTTTGGAAACGGTGCAGAGGCTCCTTCCAGTGCTGTGCCAAGCGCACGGTCTCACTCCGGAACAAGTGGTCGCAATCGCCTCCAACATTGGCGGGAAACAGGCACTCGAGACTGTCCAGCGCCTGCTTCCCGTGCTGTGCCAAGCGCACGGTCTCACTCCGGAACAAGTGGTCGCAATCGCCTCCAACATTGGCGGGAAACAGGCACTCGAGACTGTCCAGCGCCTGCTTCCCGTGCTGTGCCAAGCGCACGGTCTGACCCCAGAGCAGGTCGTGGCGATCGCAAGCCACGACGGAGGAAAGCAAGCCTTGGAAACAGTACAGAGGCTGTTGCCTGTGCTTTGTCAGGCACACGGCCTGACCCCAGAGCAGGTCGTGGCCATTGCCTCGAATGGAGGGGGCAAACAGGCGTTGGAAACCGTACAACGATTGCTGCCGGTGCTTTGTCAGGCACACGGCCTCACTCCGGAACAAGTGGTCGCAATCGCGAGCAATAACGGCGGAAAACAGGCTTTGGAAACGGTGCAGAGGCTCCTTCCAGTGCTGTGCCAAGCGCACGGTCTGACCCCAGAGCAGGTCGTGGCGATCGCAAGCCACGACGGAGGAAAGCAAGCCTTGGAAACAGTACAGAGGCTGTTGCCTGTGCTTTGTCAGGCACACGGCCTCACTCCGGAACAAGTGGTCGCAATCGCCTCCAACATTGGCGGGAAACAGGCACTCGAGACTGTCCAGCGCCTGCTTCCCGTGCTGTGCCAAGCGCACGGTCTCACTCCGGAACAAGTGGTCGCAATCGCCTCCAACATTGGCGGGAAACAGGCACTCGAGACTGTCCAGCGCCTGCTTCCCGTGCTGTGCCAAGCGCACGGTCTGACCCCAGAGCAGGTCGTGGCCATTGCCTCGAATGGAGGGGGCAAACAGGCGTTGGAAACCGTACAACGATTGCTGCCGGTGCTTTGTCAGGCACACGGCCTCACTCCGGAACAAGTGGTCGCAATCGCGAGCAATAACGGCGGAAAACAGGCTTTGGAAACGGTGCAGAGGCTCCTTCCAGTGCTGTGCCAAGCGCACGGTCTGACCCCAGAGCAGGTCGTGGCGATCGCAAGCCACGACGGAGGAAAGCAAGCCTTGGAAACAGTACAGAGGCTGTTGCCTGTGCTTTGTCAGGCACACGGCCTCACTCCGGAACAAGTGGTCGCAATCGCGAGCAATAACGGCGGAAAACAGGCTTTGGAAACGGTGCAGAGGCTCCTTCCAGTGCTGTGCCAAGCGCACGGTCTCACTCCGGAACAAGTGGTCGCAATCGCCTCCAACATTGGCGGGAAACAGGCACTCGAGACTGTCCAGCGCCTGCTTCCCGTGCTGTGCCAAGCGCACGGTCTGACCCCAGAGCAGGTCGTGGCGATCGCAAGCCACGACGGAGGAAAGCAAGCCTTGGAAACAGTACAGAGGCTGTTGCCTGTGCTTTGTCAGGCACACGGCCTGACCCCAGAGCAGGTCGTGGCGATCGCAAGCCACGACGGAGGAAAGCAAGCCTTGGAAACAGTACAGAGGCTGTTGCCTGTGCTTTGTCAGGCACACGGCCTCACTCCGGAACAAGTGGTCGCAATCGCCTCCAACATTGGCGGGAAACAGGCACTCGAGACTGTCCAGCGCCTGCTTCCCGTGCTGTGCCAAGCGCACGGT

**TALE-R: TTCCACCAGCAGCAG**

CTGACCCCAGAGCAGGTCGTGGCCATTGCCTCGAATGGAGGGGGCAAACAGGCGTTGGAAACCGTACAACGATTGCTGCCGGTGCTTTGTCAGGCACACGGCCTGACCCCAGAGCAGGTCGTGGCCATTGCCTCGAATGGAGGGGGCAAACAGGCGTTGGAAACCGTACAACGATTGCTGCCGGTGCTTTGTCAGGCACACGGCCTGACCCCAGAGCAGGTCGTGGCGATCGCAAGCCACGACGGAGGAAAGCAAGCCTTGGAAACAGTACAGAGGCTGTTGCCTGTGCTTTGTCAGGCACACGGCCTGACCCCAGAGCAGGTCGTGGCGATCGCAAGCCACGACGGAGGAAAGCAAGCCTTGGAAACAGTACAGAGGCTGTTGCCTGTGCTTTGTCAGGCACACGGCCTCACTCCGGAACAAGTGGTCGCAATCGCCTCCAACATTGGCGGGAAACAGGCACTCGAGACTGTCCAGCGCCTGCTTCCCGTGCTGTGCCAAGCGCACGGTCTGACCCCAGAGCAGGTCGTGGCGATCGCAAGCCACGACGGAGGAAAGCAAGCCTTGGAAACAGTACAGAGGCTGTTGCCTGTGCTTTGTCAGGCACACGGCCTGACCCCAGAGCAGGTCGTGGCGATCGCAAGCCACGACGGAGGAAAGCAAGCCTTGGAAACAGTACAGAGGCTGTTGCCTGTGCTTTGTCAGGCACACGGCCTCACTCCGGAACAAGTGGTCGCAATCGCCTCCAACATTGGCGGGAAACAGGCACTCGAGACTGTCCAGCGCCTGCTTCCCGTGCTGTGCCAAGCGCACGGTCTCACTCCGGAACAAGTGGTCGCAATCGCGAGCAATAACGGCGGAAAACAGGCTTTGGAAACGGTGCAGAGGCTCCTTCCAGTGCTGTGCCAAGCGCACGGTCTGACCCCAGAGCAGGTCGTGGCGATCGCAAGCCACGACGGAGGAAAGCAAGCCTTGGAAACAGTACAGAGGCTGTTGCCTGTGCTTTGTCAGGCACACGGCCTCACTCCGGAACAAGTGGTCGCAATCGCCTCCAACATTGGCGGGAAACAGGCACTCGAGACTGTCCAGCGCCTGCTTCCCGTGCTGTGCCAAGCGCACGGTCTCACTCCGGAACAAGTGGTCGCAATCGCGAGCAATAACGGCGGAAAACAGGCTTTGGAAACGGTGCAGAGGCTCCTTCCAGTGCTGTGCCAAGCGCACGGTCTGACCCCAGAGCAGGTCGTGGCGATCGCAAGCCACGACGGAGGAAAGCAAGCCTTGGAAACAGTACAGAGGCTGTTGCCTGTGCTTTGTCAGGCACACGGCCTCACTCCGGAACAAGTGGTCGCAATCGCCTCCAACATTGGCGGGAAACAGGCACTCGAGACTGTCCAGCGCCTGCTTCCCGTGCTGTGCCAAGCGCACGGTCTCACTCCGGAACAAGTGGTCGCAATCGCGAGCAATAACGGCGGAAAACAGGCTTTGGAAACGGTGCAGAGGCTCCTTCCAGTGCTGTGCCAAGCGCACGGT

**The sequence of pL20 and pR16**

pL20 (…..-UBiqutin -3xFlag-NLS-N’-[TALE-L]-1/2T-C’-FokI-nosT-…..)

GCTGCTTCGCGATGTACGGGCCAGATATACAAGCTTGCATGCCTGCAGTGCAGCGTGACCCGGTCGTGCCCCTCTCTAGAGATAATGAGCATTGCATGTCTAAGTTATAAAAAATTACCACATATTTTTTTTGTCACACTTGTTTGAAGTGCAGTTTATCTATCTTTATACATATATTTAAACTTTACTCTACGAATAATATAATCTATAGTACTACAATAATATCAGTGTTTTAGAGAATCATATAAATGAACAGTTAGACATGGTCTAAAGGACAATTGAGTATTTTGACAACAGGACTCTACAGTTTTATCTTTTTAGTGTGCATGTGTTCTCCTTTTTTTTTGCAAATAGCTTCACCTATATAATACTTCATCCATTTTATTAGTACATCCATTTAGGGTTTAGGGTTAATGGTTTTTATAGACTAATTTTTTTAGTACATCTATTTTATTCTATTTTAGCCTCTAAATTAAGAAAACTAAAACTCTATTTTAGTTTTTTTATTTAATAATTTAGATATAAAATAGAATAAAATAAAGTGACTAAAAATTAAACAAATACCCTTTAAGAAATTAAAAAAACTAAGGAAACATTTTTCTTGTTTCGAGTAGATAATGCCAGCCTGTTAAACGCCGTCGACGAGTCTAACGGACACCAACCAGCGAACCAGCAGCGTCGCGTCGGGCCAAGCGAAGCAGACGGCACGGCATCTCTGTCGCTGCCTCTGGACCCCTCTCGAGAGTTCCGCTCCACCGTTGGACTTGCTCCGCTGTCGGCATCCAGAAATTGCGTGGCGGAGCGGCAGACGTGAGCCGGCACGGCAGGCGGCCTCCTCCTCCTCTCACGGCACCGGCAGCTACGGGGGATTCCTTTCCCACCGCTCCTTCGCTTTCCCTTCCTCGCCCGCCGTAATAAATAGACACCCCCTCCACACCCTCTTTCCCCAACCTCGTGTTGTTCGGAGCGCACACACACACAACCAGATCTCCCCCAAATCCACCCGTCGGCACCTCCGCTTCAAGGTACGCCGCTCGTCCTCCCCCCCCCCCCCTCTCTACCTTCTCTAGATCGGCGTTCCGGTCCATGGTTAGGGCCCGGTAGTTCTACTTCTGTTCATGTTTGTGTTAGATCCGTGTTTGTGTTAGATCCGTGCTGCTAGCGTTCGTACACGGATGCGACCTGTACGTCAGACACGTTCTGATTGCTAACTTGCCAGTGTTTCTCTTTGGGGAATCCTGGGATGGCTCTAGCCGTTCCGCAGACGGGATCGATTTCATGATTTTTTTTGTTTCGTTGCATAGGGTTTGGTTTGCCCTTTTCCTTTATTTCAATATATGCCGTGCACTTGTTTGTCGGGTCATCTTTTCATGCTTTTTTTTGTCTTGGTTGTGATGATGTGGTCTGGTTGGGCGGTCGTTCTAGATCGGAGTAGAATTCTGTTTCAAACTACCTGGTGGATTTATTAATTTTGGATCTGTATGTGTGTGCCATACATATTCATAGTTACGAATTGAAGATGATGGATGGAAATATCGATCTAGGATAGGTATACATGTTGATGCGGGTTTTACTGATGCATATACAGAGATGCTTTTTGTTCGCTTGGTTGTGATGATGTGGTGTGGTTGGGCGGTCGTTCATTCGTTCTAGATCGGAGTAGAATACTGTTTCAAACTACCTGGTGTATTTATTAATTTTGGAACTGTATGTGTGTGTCATACATCTTCATAGTTACGAGTTTAAGATGGATGGAAATATCGATCTAGGATAGGTATACATGTTGATGTGGGTTTTACTGATGCATATACATGATGGCATATGCAGCATCTATTCATATGCTCTAACCTTGAGTACCTATCTATTATAATAAACAAGTATGTTTTATAATTATTTTGATCTTGATATACTTGGATGATGGCATATGCAGCAGCTATATGTGGATTTTTTTAGCCCTGCCTTCATACGCTATTTATTTGCTTGGTACTGTTTCTTTTGTCGATGCTCACCCTGTTGTTTGGTGTTACTTCTGCAGACTAGTCCAGTGTGGTGGAATTCGCCATGGACTACAAAGACCATGACGGTGATTATAAAGATCATGACATCGATTACAAGGATGACGATGACAAGATGGCCCCCAAGAAGAAGAGGAAGGTGGGCATCCACGGGGTACCCATGGTAGATTTGAGAACTTTGGGATATTCACAGCAGCAGCAGGAAAAGATCAAGCCCAAAGTGAGGTCGACAGTCGCGCAGCATCACGAAGCGCTGGTGGGTCATGGGTTTACACATGCCCACATCGTAGCCTTGTCGCAGCACCCTGCAGCCCTTGGCACGGTCGCCGTCAAGTACCAGGACATGATTGCGGCGTTGCCGGAAGCCACACATGAGGCGATCGTCGGTGTGGGGAAACAGTGGAGCGGAGCCCGAGCGCTTGAGGCCCTGTTGACGGTCGCGGGAGAGCTGAGAGGGCCTCCCCTTCAGCTGGACACGGGCCAGTTGCTGAAGATCGCGAAGCGGGGAGGAGTCACGGCGGTCGAGGCGGTGCACGCGTGGCGCAATGCGCTCACGGGAGCACCCCTCAAC**[TALE-L]**CTCACGCCTGAGCAGGTAGTGGCTATTGCATCCAATGGAGGGGGCAGACCCGCACTGGAGTCAATCGTGGCCCAGCTTTCGAGGCCGGACCCCGCGCTGGCCGCACTCACTAATGATCATCTTGTAGCGCTGGCCTGCCTCGGCGGACGACCCGCCTTGGATGCGGTGAAGAAGGGGCTCCCGCACGCGCCTGCATTGATTAAGCGGACCAACAGAAGGATTCCCGAGAGGACATCACATCGAGTGGCAGGATCCCAGCTGGTGAAGAGCGAGCTGGAGGAGAAGAAGTCCGAGCTGCGGCACAAGCTGAAGTACGTGCCCCACGAGTACATCGAGCTGATCGAGATCGCCAGGAACAGCACCCAGGACCGCATCCTGGAGATGAAGGTGATGGAGTTCTTCATGAAGGTGTACGGCTACAGGGGAAAGCACCTGGGCGGAAGCAGAAAGCCTGACGGCGCCATCTATACAGTGGGCAGCCCCATCGATTACGGCGTGATCGTGGACACAAAGGCCTACAGCGGCGGCTACAATCTGCCTATCGGCCAGGCCGACGAGATGGAGAGATACGTGGAGGAGAACCAGACCCGGAATAAGCACCTCAACCCCAACGAGTGGTGGAAGGTGTACCCTAGCAGCGTGACCGAGTTCAAGTTCCTGTTCGTGAGCGGCCACTTCAAGGGCAACTACAAGGCCCAGCTGACCAGGCTGAACCACATCACCAACTGCAATGGCGCCGTGCTGAGCGTGGAGGAGCTGCTGATCGGCGGCGAGATGATCAAAGCCGGCACCCTGACACTGGAGGAGGTGCGGCGCAAGTTCAACAACGGCGAGATCAACTTCAGATCTTGATAACTCGAGCTCGAGGATCGTTCAAACATTTGGCAATAAAGTTTCTTAAGATTGAATCCTGTTGCCGGTCTTGCGATGATTATCATATAATTTCTGTTGAATTACGTTAAGCATGTAATAATTAACATGTAATGCATGACGTTATTTATGAGATGGGTTTTTATGATTAGAGTCCCGCAATTATACATTTAATACGCGATAGAAAACAAAATATAGCGCGCAAACTAGGATAAATTATCGCGCGCGGTGTCATCTATGTTACTAGATCCGATGATAAGCTGTCAAACATGAGAATTGGCGCGCCGTTTTATGGACAGCAAGCGAACCGGAATTGCCAGCTGGGGCGCCCTCTGGTAAGGTTGGGAAGCCCTGCAAAGTAAACTGGATGGCTTTCTTGCCGCCAAGGATCTGATGGCGCAGGGGATCAAGCTCTGATCAAGAGACAGGATGAGGATCGTTTCGCATGATTGAACAAGATGGATTGCACGCAGGTTCTCCGGCCGCTTGGGTGGAGAGGCTATTCGGCTATGACTGGGCACAACAGACAATCGGCTGCTCTGATGCCGCCGTGTTCCGGCTGTCAGCGCAGGGGCGCCCGGTTCTTTTTGTCAAGACCGACCTGTCCGGTGCCCTGAATGAACTGCAAGACGAGGCAGCGCGGCTATCGTGGCTGGCCACGACGGGCGTTCCTTGCGCAGCTGTGCTCGACGTTGTCACTGAAGCGGGAAGGGACTGGCTGCTATTGGGCGAAGTGCCGGGGCAGGATCTCCTGTCATCTCACCTTGCTCCTGCCGAGAAAGTATCCATCATGGCTGATGCAATGCGGCGGCTGCATACGCTTGATCCGGCTACCTGCCCATTCGACCACCAAGCGAAACATCGCATCGAGCGAGCACGTACTCGGATGGAAGCCGGTCTTGTCGATCAGGATGATCTGGACGAAGAGCATCACGGGCTCGCGCCAGCCGAACTGTTCGCCAGGCTCAAGGCGAGCATGCCCGACGGCGAGGATCTCGTCGTGACCCATGGCGATGCCTGCTTGCCGAATATCATGGTGGAAAATGGCCGCTTTTCTGGATTCATCGACTGTGCCGGCTGGTGTGGCGGACCGCTATCAGGACATAGCGTTGGCTACCGTGATATTGCTGAAGAGCTTGCGCGAATGGCTGACGCTTCTCGTGCTTACGGTATCGCCGCCTCCCGATGCATCAGTGCACTTTTCGGGGAAATGTGCGCGACCCTATTTGTTTATTTTTCTAATACATCAAATATGTATCCGCTCATGAGACAATAACCTGATAAATGCTTCAATAATAGCACGTGCTAAAACTTCATTTTTAAATTTAAAAGGATCTAGGTGAAGATCCTTTTTGATAATCTCATGACCAAAATCCTTTAACGTGAGTTTTCGTTCCACTGAGCGTCAGACCCCGTAGAAAAGATCAAAGGATCTTCTTGAGATCCTTTTTTTCTGCGCGTAATCTGCTGCTTGCAAACAAAAAAACCACCGCTACCAGCGGTGGTTTGTTTGCCGGATCAAGAGCTACCAACTCTTTTTCCGAAGGTAACTGGCTTCAGCAGAGCGCAGATACCAAATACTGTTCTTCTAGTGTAGCCGTAGTTAGGCCACCACTTCAAGAACTCTGTAGCACCGCCTACATACCTCGCTCTGCTAATCCTGTTACCAGTGGCTGCTGCCAGTGGCGATAAGTCGTGTCTTACCGGGTTGGACTCAAGACGATAGTTACCGGATAAGGCGCAGCGGTCGGGCTGAACGGGGGGTTCGTGCACACAGCCCAGCTTGGAGCGAACGACCTACACCGAACTGAGATACCTACAGCGTGAGCTATGAGAAAGCGCCACGCTTCCCGAAGGGAGAAAGGCGGACAGGTATCCGGTAAGCGGCAGGGTCGGAACAGGAGAGCGCACGAGGGAGCTTCCAGGGGGAAACGCCTGGTATCTTTATAGTCCTGTCGGGTTTCGCCACCTCTGACTTGAGCGTCGATTTTTGTGATGCTCGTCAGGGGGGCGGAGCCTATGGAAAAACGCCAGCAACGCGGCCTTTTTACGGTTCCTGGCCTTTTGCTGGCCTTTTGCTCACATGTTCTT

pR16 (…..-35S -3xFlag-NLS-N’-[TALE-R]-1/2T-C’-FokI-nosT-…..)

GCTGCTTCGCGATGTACGGGCCAGATATACGGCGCGCCATGGTGGAGCACGACACTCTCGTCTACTCCAAGAATATCAAAGATACAGTCTCAGAAGACCAAAGGGCTATTGAGACTTTTCAACAAAGGGTAATATCGGGAAACCTCCTCGGATTCCATTGCCCAGCTATCTGTCACTTCATCAAAAGGACAGTAGAAAAGGAAGGTGGCACCTACAAATGCCATCATTGCGATAAAGGAAAGGCTATCGTTCAAGATGCCTCTGCCGACAGTGGTCCCAAAGATGGACCCCCACCCACGAGGAGCATCGTGGAAAAAGAAGACGTTCCAACCACGTCTTCAAAGCAAGTGGATTGATGTGATAACATGGTGGAGCACGACACTCTCGTCTACTCCAAGAATATCAAAGATACAGTCTCAGAAGACCAAAGGGCTATTGAGACTTTTCAACAAAGGGTAATATCGGGAAACCTCCTCGGATTCCATTGCCCAGCTATCTGTCACTTCATCAAAAGGACAGTAGAAAAGGAAGGTGGCACCTACAAATGCCATCATTGCGATAAAGGAAAGGCTATCGTTCAAGATGCCTCTGCCGACAGTGGTCCCAAAGATGGACCCCCACCCACGAGGAGCATCGTGGAAAAAGAAGACGTTCCAACCACGTCTTCAAAGCAAGTGGATTGATGTGATATCTCCACTGACGTAAGGGATGACGCACAATCCCACTATCCTTCGCAAGACCTTCCTCTATATAAGGAAGTTCATTTCATTTGGAGAGGACACGCTGAAATCACCAGTCTCTCTCTACAAATCTATCTCTGCTAGCGTTTAAACTTAAGCTGATCCACTAGTCCAGTGTGGTGGAATTCGCCATGGACTACAAAGACCATGACGGTGATTATAAAGATCATGACATCGATTACAAGGATGACGATGACAAGATGGCCCCCAAGAAGAAGAGGAAGGTGGGCATCCACGGGGTACCCATGGTAGATTTGAGAACTTTGGGATATTCACAGCAGCAGCAGGAAAAGATCAAGCCCAAAGTGAGGTCGACAGTCGCGCAGCATCACGAAGCGCTGGTGGGTCATGGGTTTACACATGCCCACATCGTAGCCTTGTCGCAGCACCCTGCAGCCCTTGGCACGGTCGCCGTCAAGTACCAGGACATGATTGCGGCGTTGCCGGAAGCCACACATGAGGCGATCGTCGGTGTGGGGAAACAGTGGAGCGGAGCCCGAGCGCTTGAGGCCCTGTTGACGGTCGCGGGAGAGCTGAGAGGGCCTCCCCTTCAGCTGGACACGGGCCAGTTGCTGAAGATCGCGAAGCGGGGAGGAGTCACGGCGGTCGAGGCGGTGCACGCGTGGCGCAATGCGCTCACGGGAGCACCCCTCAAC**[TALE-R]**CTCACGCCTGAGCAGGTAGTGGCTATTGCATCCAATGGAGGGGGCAGACCCGCACTGGAGTCAATCGTGGCCCAGCTTTCGAGGCCGGACCCCGCGCTGGCCGCACTCACTAATGATCATCTTGTAGCGCTGGCCTGCCTCGGCGGACGACCCGCCTTGGATGCGGTGAAGAAGGGGCTCCCGCACGCGCCTGCATTGATTAAGCGGACCAACAGAAGGATTCCCGAGAGGACATCACATCGAGTGGCAGGATCCCAGCTGGTGAAGAGCGAGCTGGAGGAGAAGAAGTCCGAGCTGCGGCACAAGCTGAAGTACGTGCCCCACGAGTACATCGAGCTGATCGAGATCGCCAGGAACAGCACCCAGGACCGCATCCTGGAGATGAAGGTGATGGAGTTCTTCATGAAGGTGTACGGCTACAGGGGAAAGCACCTGGGCGGAAGCAGAAAGCCTGACGGCGCCATCTATACAGTGGGCAGCCCCATCGATTACGGCGTGATCGTGGACACAAAGGCCTACAGCGGCGGCTACAATCTGCCTATCGGCCAGGCCGACGAGATGCAGAGATACGTGAAGGAGAACCAGACCCGGAATAAGCACATCAACCCCAACGAGTGGTGGAAGGTGTACCCTAGCAGCGTGACCGAGTTCAAGTTCCTGTTCGTGAGCGGCCACTTCAAGGGCAACTACAAGGCCCAGCTGACCAGGCTGAACCACAAAACCAACTGCAATGGCGCCGTGCTGAGCGTGGAGGAGCTGCTGATCGGCGGCGAGATGATCAAAGCCGGCACCCTGACACTGGAGGAGGTGCGGCGCAAGTTCAACAACGGCGAGATCAACTTCTGATAAGAGCTCTCGACAAGCTCGAGTTTCTCCATAATAATGTGTGAGTAGTTCCCAGATAAGGGAATTAGGGTTCCTATAGGGTTTCGCTCATGTGTTGAGCATATAAGAAACCCTTAGTATGTATTTGTATTTGTAAAATACTTCTATCAATAAAATTTCTAATTCCTAAAACCAAAATCCAGTACTAAAATCCAGATCCCCCGAATTAATTCGGCGTTAATTCAGGCGGCCGCCTTCTACTGGGCGGTTTTATGGACAGCAAGCGAACCGGAATTGCCAGCTGGGGCGCCCTCTGGTAAGGTTGGGAAGCCCTGCAAAGTAAACTGGATGGCTTTCTTGCCGCCAAGGATCTGATGGCGCAGGGGATCAAGCTCTGATCAAGAGACAGGATGAGGATCGTTTCGCATGATTGAACAAGATGGATTGCACGCAGGTTCTCCGGCCGCTTGGGTGGAGAGGCTATTCGGCTATGACTGGGCACAACAGACAATCGGCTGCTCTGATGCCGCCGTGTTCCGGCTGTCAGCGCAGGGGCGCCCGGTTCTTTTTGTCAAGACCGACCTGTCCGGTGCCCTGAATGAACTGCAAGACGAGGCAGCGCGGCTATCGTGGCTGGCCACGACGGGCGTTCCTTGCGCAGCTGTGCTCGACGTTGTCACTGAAGCGGGAAGGGACTGGCTGCTATTGGGCGAAGTGCCGGGGCAGGATCTCCTGTCATCTCACCTTGCTCCTGCCGAGAAAGTATCCATCATGGCTGATGCAATGCGGCGGCTGCATACGCTTGATCCGGCTACCTGCCCATTCGACCACCAAGCGAAACATCGCATCGAGCGAGCACGTACTCGGATGGAAGCCGGTCTTGTCGATCAGGATGATCTGGACGAAGAGCATCACGGGCTCGCGCCAGCCGAACTGTTCGCCAGGCTCAAGGCGAGCATGCCCGACGGCGAGGATCTCGTCGTGACCCATGGCGATGCCTGCTTGCCGAATATCATGGTGGAAAATGGCCGCTTTTCTGGATTCATCGACTGTGCCGGCTGGTGTGGCGGACCGCTATCAGGACATAGCGTTGGCTACCGTGATATTGCTGAAGAGCTTGCGCGAATGGCTGACGCTTCTCGTGCTTACGGTATCGCCGCCTCCCGATGCATCAGTGCACTTTTCGGGGAAATGTGCGCGACCCTATTTGTTTATTTTTCTAATACATCAAATATGTATCCGCTCATGAGACAATAACCTGATAAATGCTTCAATAATAGCACGTGCTAAAACTTCATTTTTAAATTTAAAAGGATCTAGGTGAAGATCCTTTTTGATAATCTCATGACCAAAATCCTTTAACGTGAGTTTTCGTTCCACTGAGCGTCAGACCCCGTAGAAAAGATCAAAGGATCTTCTTGAGATCCTTTTTTTCTGCGCGTAATCTGCTGCTTGCAAACAAAAAAACCACCGCTACCAGCGGTGGTTTGTTTGCCGGATCAAGAGCTACCAACTCTTTTTCCGAAGGTAACTGGCTTCAGCAGAGCGCAGATACCAAATACTGTTCTTCTAGTGTAGCCGTAGTTAGGCCACCACTTCAAGAACTCTGTAGCACCGCCTACATACCTCGCTCTGCTAATCCTGTTACCAGTGGCTGCTGCCAGTGGCGATAAGTCGTGTCTTACCGGGTTGGACTCAAGACGATAGTTACCGGATAAGGCGCAGCGGTCGGGCTGAACGGGGGGTTCGTGCACACAGCCCAGCTTGGAGCGAACGACCTACACCGAACTGAGATACCTACAGCGTGAGCTATGAGAAAGCGCCACGCTTCCCGAAGGGAGAAAGGCGGACAGGTATCCGGTAAGCGGCAGGGTCGGAACAGGAGAGCGCACGAGGGAGCTTCCAGGGGGAAACGCCTGGTATCTTTATAGTCCTGTCGGGTTTCGCCACCTCTGACTTGAGCGTCGATTTTTGTGATGCTCGTCAGGGGGGCGGAGCCTATGGAAAAACGCCAGCAACGCGGCCTTTTTACGGTTCCTGGCCTTTTGCTGGCCTTTTGCTCACATGTTCTT

**The 898 bp-homologous fragment contained in the HRP (pMD-EPSPM).** The underline indicates the binding sites of primers EMS and EMR, the box indicates the substituted base (C-T):

ATTATGCAATTTTGAGGCTCCTCTACATCATTATAATTCCCCAATACATTGCTCTTTATTCTTAATAGCTTTGATCGCGAAATTTAACATTTTAATTCTTGAGCTGTTATTTTGTAGCATCAGTTTATCATGAGCCATGTTTGGTACTAAATATACAATCCCTTGGGTTTATTTGTTTCCAAGCATGTCATTAACTTATCTTAATGTGGACAAGAAACTGATGCCTGCTTACATTGCTATTATTTCAAGCGGGTATTGATCCTTTGACATGTGATTGATCATTTTTTTTTCTCTGGTTATTAGGGCACAACAGTGGTGGACAACTTGCTGAACAGTGAGGATGTTCACTACATGCTTGAGGCCCTGAAAGCCCTCGGGCTCTCTGTGGAAGCAGATAAAGTTGCAAAAAGAGCTGTAGTCGTTGGCTGTGGTGGCAAGTTTCCTGTTGAGAAGGATGCGAAAGAGGAAGTGCAACTCTTCTTGGGGAACGCTGGAACTGCAATGCGACTATTGACAGCAGCCGTGACTGCTGCTGGTGGAAATGCAACGTATGTTTTTTTTTTTAATGTTTATGAAAATATGTATGGAATTCATGGGGTATGTTTTATGACCTTTTTCTTTACCATCAGTTATGTGCTTGATGGAGTGCCACGAATGAGGGAGAGACCGATTGGTGACTTGGTTGTCGGGTTGAAACAACTTGGTGCGGATGTCGACTGTTTCCTTGGCACTGAATGCCCACCTGTTCGTGTCAAGGGAATTGGAGGACTTCCTGGTGGCAAGGTTAGTTACTCCTAAACTGCATCCTTTGTACTTCTGTATGCACCTCAATTCTTTGTCAACCTTCTGCATTTATAAGGAACATTCTATGATGCAATTCGACCTTACACTGCACAGT
